# Spt6 is a maintenance factor for centromeric CENP-A

**DOI:** 10.1038/s41467-020-16695-7

**Published:** 2020-06-10

**Authors:** Georg O. M. Bobkov, Anming Huang, Sebastiaan J. W. van den Berg, Sreyoshi Mitra, Eduard Anselm, Vasiliki Lazou, Sarah Schunter, Regina Feederle, Axel Imhof, Alexandra Lusser, Lars E. T. Jansen, Patrick Heun

**Affiliations:** 1 Wellcome Centre for Cell Biology, School of Biological Sciences, The University of Edinburgh, Edinburgh, EH9 3BF UK; 2 Faculty of Biology, Albert-Ludwigs-Universität Freiburg, 79104 Freiburg, Germany; 3 Institute of Molecular Biology, Biocenter, Medical University of Innsbruck, Innrain 80-82, A-6020 Innsbruck, Austria; 4 Instituto Gulbenkian de Ciência, 2780-156 Oeiras, Portugal; 5 Department of Biochemistry, University of Oxford, South Parks Road, Oxford, OX1 3QU UK; 6 Molecular Biology Division, Biomedical Center, Faculty of Medicine, LMU, Munich, Germany; 7 Monoclonal Antibody Core Facility, Helmholtz Zentrum München, German Research Center for Environmental Health (GmbH), 85764 Neuherberg, Germany; 8 BioMedical Center and Center for Integrated Protein Sciences Munich, Ludwig-Maximilians-University of Munich, Großhaderner Straße 9, 82152 Planegg-Martinsried, Germany

**Keywords:** Centromeres, Epigenetics, Transcription

## Abstract

Replication and transcription of genomic DNA requires partial disassembly of nucleosomes to allow progression of polymerases. This presents both an opportunity to remodel the underlying chromatin and a danger of losing epigenetic information. Centromeric transcription is required for stable incorporation of the centromere-specific histone dCENP-A in M/G1 phase, which depends on the eviction of previously deposited H3/H3.3-placeholder nucleosomes. Here we demonstrate that the histone chaperone and transcription elongation factor Spt6 spatially and temporarily coincides with centromeric transcription and prevents the loss of old CENP-A nucleosomes in both *Drosophila* and human cells. Spt6 binds directly to dCENP-A and dCENP-A mutants carrying phosphomimetic residues alleviate this association. Retention of phosphomimetic dCENP-A mutants is reduced relative to wildtype, while non-phosphorylatable dCENP-A retention is increased and accumulates at the centromere. We conclude that Spt6 acts as a conserved CENP-A maintenance factor that ensures long-term stability of epigenetic centromere identity during transcription-mediated chromatin remodeling.

## Abstract (web summary)

CENP-A is a stable centromere mark, although active transcription poses a potential threat for retaining CENP-A through chromatin remodeling and nucleosome eviction. Here, the authors show that maintenance of the centromeric mark is preserved by Spt6, which recycles CENP-A nucleosomes.

## Introduction

Centromeres constitute a platform for the assembly of the kinetochore during mitosis and mediate the attachment of chromosomes to the mitotic spindle for proper segregation of chromosomes. The position of the centromere is mostly determined epigenetically through the incorporation of the H3-variant CENP-A (CID/dCENP-A in *Drosophila*)^1,2^. Centromeric chromatin is composed of interspersed arrays of CENP-A and canonical histone H3 nucleosomes^3^. While canonical H3 is replenished during DNA replication in S-phase^4^, loading of CENP-A in *Drosophila* and humans takes place in a replication-independent manner from late mitosis to G1^5–9^. This process requires the exchange or removal of so-called placeholder nucleosomes containing H3 and H3.3, which have been positioned on centromeric DNA-sequences during the previous S-phase^10,11^.

As expected for an epigenetic mark, centromeric CENP-A nucleosomes are remarkably stable and can be propagated not only over multiple cell divisions but also across generations. Indeed, epitope-tag labeling of dCENP-A revealed that once fully incorporated, CENP-A turnover in healthy proliferating cells is almost exclusively restricted to replicative dilution^12,13^. Some of this stability is conferred to CENP-A by other centromere factors that act on the intact DNA-bound nucleosome itself. While CENP-C reshapes and clamps down the CENP-A nucleosome, CENP-N helps fastening CENP-A to the underlying DNA^14,15^.The remarkable stability of CENP-A is further demonstrated by the fact that CENP-A nucleosomes that are assembled in mouse oocytes before birth, persist in the chromatin of prophase I-arrested cells for over a year and are sufficient for genome transmission to embryos through the entire fertile lifespan of the mouse^16^.

In actively dividing cells, however, chromatin is a highly dynamic structure. Cellular processes that require direct DNA contact like DNA replication or transcription induce large-scale chromatin remodeling events to allow the progression of DNA- and RNA- polymerases. This involves partial or full disassembly of nucleosomes^17^, which challenges the stable transmission of epigenetic marks encoded in histone variants or histone tail modifications. Accordingly, mechanisms need to be in place to ensure faithful transmission of epigenetic signals during replication and transcription.

CENP-A is the key epigenetic mark for the centromere and has been shown to be maintained during the replication of centromeric DNA^5,6,12^. Recent work identified the MCM2-7 replicative helicase to recycle previously deposited H3/H4, H3.3/H4, and CENP-A/H4 tetramers together with other chaperones during S-phase to ensure the transfer of parental nucleosomes to freshly replicated DNA^18–21^.

Centromeres are also sites of active transcription, as revealed by the centromeric presence of RNA Polymerase II (RNAPII), centromeric RNA transcripts and transcription-associated histone modifications in various organisms including yeast, flies and humans^9,22–31^. Centromeric transcription is important for centromere function^32^, and it has been proposed that transcription-mediated chromatin remodeling is required for CENP-A loading^9,22,33^. However, it is currently unclear how old CENP-A nucleosomes survive the passage of the elongating RNAPII. Active removal of CENP-A through induced upregulation of transcription at the centromere has been observed in a variety of organism including on plasmids in budding yeast, on artificial chromosomes in human cells^34,35^ and as a consequence of genotoxic stress in senescent murine cells^36^.

To counteract the transcription-coupled eviction of nucleosomes and to ensure genome integrity, chromatin needs to be rapidly re-established in the wake of the DNA- and RNA polymerase. During DNA replication, this is achieved through deposition of canonical histones, whereas nucleosome gaps created by genomic transcription are filled through the replication-independent incorporation of H3.3^4,37^ as well as the recycling of displaced old histones. Disassembly of nucleosomes in front of a progressing RNAPII involves the histone chaperone Facilitates Chromatin Transcription (FACT)^17,18^. FACT also acts to reassemble nucleosomes behind RNAPII together with the transcription elongation factor and histone chaperone Spt6^38^. Spt6 can interact with histones, assembles them into nucleosomes^39^, and is able to increase the elongation rate of RNAPII both in vitro and in vivo^40,41^.

While a role for FACT at the centromere and its importance for CENP-A deposition has already been demonstrated in numerous organisms^22,33,42,43^, little is known about a centromeric function of Spt6. Interestingly, Spt6 was detected in a CENP-A pull-down and mass-spectrometry experiment both in budding yeast and in flies^44,45^. Budding yeast mutants of Spt6 further show segregation defects for a chromosome fragment^46^, whereas mutants in *Schizosaccharomyces pombe* exhibit genome-wide CENP-A misincorporation^22^. Importantly, Spt6 prevents transcription-coupled loss of nucleosomes in gene bodies by reincorporating H3/H4 tetramers displaced during transcription. Consequently, Spt6 preserves the epigenetic information encoded in histone tail posttranslational modifications (PTMs) of recycled nucleosomes^38,47^. Spt6 further stabilizes nucleosomes by removing transcription-induced histone acetylation through a self-enforcing protein network comprising Spt6, the histone deacetylase Rpd3 and the H3K36 methyl-transferase Set2^48,49^. Consistent with a major role of Spt6 in the restoration of transcriptionally perturbed chromatin in the wake of a progressing RNAPII, cryptic promoters are activated within transcription units in mutants for Spt6^50,51^.

Here we demonstrate that transcription at the centromere, while being important for loading of new CENP-A, simultaneously poses a threat to the maintenance of ancestral CENP-A nucleosomes. We further show that long-term stability of the centromeric mark is achieved through effective recycling of expelled dCENP-A by Spt6 in both *Drosophila* and in human cells.

## Results

### Spt6 is present at mitotic and G1 centromeres

To identify novel factors associated with *Drosophila* centromeres we previously affinity-purified GFP-tagged dCENP-A containing nucleosomes and combined it with mass-spectroscopy analysis. Among the proteins enriched in dCENP-A containing chromatin was the transcription elongation factor and histone chaperone Spt6^45^.

**Fig. 1:**
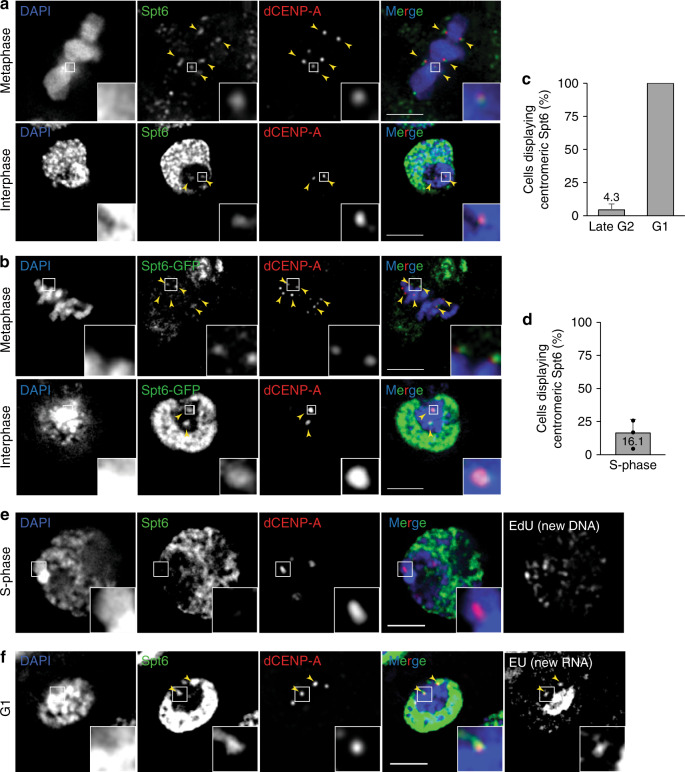
Spt6 localizes to centromeres in mitosis and G1. **a** Single optical section of fixed metaphase (upper panel) and interphase (lower panel) cells immunostained for endogenous Spt6 and dCENP-A. *N* = 3 independent experiments. **b** Single optical section of fixed metaphase (upper panel) and interphase (lower panel) S2 cells expressing Spt6-GFP. *N* = 3 independent experiments. dCENP-A immunodetection served as a marker of centromeres. **c** Analysis of the localization of GFP-tagged Spt6 using life imaging of cells 1 h before (late G2; *n* = 23 cells) and 1 h after (G1; *n* = 39 cells) anaphase onset. Movies were taken in a cumulative way. Error bars = standard error of the mean; SEM. Quantification was based on images shown in Supplementary Fig.1a. **d** Quantification of Spt6 localization in fixed EdU incorporating S-phase cells exemplified in (**e**). *N* = 3 independent experiments; *n* = 93 S-phase cells. Data are represented as mean + standard deviation; SD. **e** Single optical section of fixed S-phase cell immunostained for Spt6 and dCENP-A. Incorporation of EdU served as a marker for S-phase. Boxes indicate the 3× enlarged inset. Scale bar represents 3 µm. **f** G1 phase S2 cell with nascent RNA production labeled by click-iT EU and immunostained for Spt6. *N* = 3 independent experiments. Yellow arrow heads point to centromeric Spt6. Boxes indicate the 3× enlarged inset. Scale bars represent 3 µm. Source data are provided as a Source Data file.

To verify the identification of Spt6 as a centromere-associated protein, we investigated its cellular localization using fluorescence microscopy. Both endogenous Spt6 as well as a GFP-tagged transgenic construct were detected at mitotic centromeres (Fig. 1a, b; upper panel) and colocalised with dCENP-A and RNAPII (Supplementary Fig. 1a). In interphase, Spt6 most prominently stained non-centromeric, transcriptionally active areas, whereas the centromere surrounding heterochromatin was largely devoid of any signal. In a subpopulation of interphase cells, additional Spt6 foci that overlapped with centromere counterstaining were visible (Fig. 1a, b; lower panel). To characterize the localization of Spt6 with respect to various cell cycle stages in greater detail, we investigated cells that simultaneously expressed dCENP-A-mCherry and Spt6-GFP by live-cell microscopy. In late G2 cells just prior to the entry into mitosis, Spt6-GFP was not detectable at centromeres (Fig. 1c and Supplementary Fig. 1b). While high levels of nucleo-cytoplasmic Spt6-GFP during mitosis interfered with its precise localization in live-cell imaging, Spt6-GFP appeared at centromeres within minutes of entering the subsequent G1 phase (Fig. 1c) and remained there for a period of 3–6 h (Supplementary Fig. 1b). Analysis of fixed S-phase cells labeled through 5-ethynyl-2′-deoxyuridine EdU incorporation (click-IT^®^) revealed very little to no centromeric Spt6 (Fig. 1d, e), whereas investigation of midbody containing cells confirmed its presence at early G1 centromeres (Supplementary Fig. 1c).

This cell cycle-dependent localization pattern mirrors the previous mapping of centromeric RNAPII and centromere-associated transcripts to mitotic and G1 centromeres^9^. Indeed, combining Spt6 immunostaining with pulse labeling of nascent RNA using the Click-It^®^ technology allowed the simultaneous detection of Spt6 together with centromere-associated transcripts at the same interphase centromeres (Fig. 1f). Taken together, centromeric association of Spt6 is cell cycle regulated and largely restricted to centromeres of mitotic and G1 cells.

### RNAi-mediated depletion of Spt6 causes mitotic defects

Next, we decided to investigate the effects of Spt6 depletion via RNA interference (RNAi) in *Drosophila* S2 cells (Supplementary Fig. 2a). Interestingly, we observed a strong increase in mitotic defects in Spt6-depleted cells to levels comparable to prolonged RNAi depletion of the highly stable dCENP-A protein (Supplementary Fig. 2b). While mitotic defects in Spt6 RNAi were dominated by lagging chromosomes, dCENP-A depletion mainly resulted in chromosome congression defects (Supplementary Fig. 2c). This suggests a potential impact on centromere functionality, which is consistent with previously reported missegregation of a chromosome fragment in a budding yeast Spt6 mutant^46^. However, during the course of the RNAi experiment, we noticed an apparent decrease of mitotic cells in samples treated for more than 2 days with dsRNAs targeting Spt6 (Supplementary Fig. 2d). Measurement of cell numbers in RNAi-treated cultures confirmed that depletion of Spt6 leads to a cell cycle block whereas control cells depleted for the white protein were unaffected (Supplementary Fig. 2e). Subsequent fluorescence-activated cell sorting (FACS) analysis revealed that the cell cycle arrest of Spt6-depleted cells was not specific and instead occurred across all cell cycle stages (Supplementary Fig. 2f).

### Spt6 depletion by deGradFP reduced dCENP-A at centromeres

Spt6 localization to centromeres in mitosis and G1 matches the time window where new dCENP-A is incorporated into *Drosophila* centromeres^6–9^. Spt6 has further been shown to increase the elongation rate of RNAPII^40,41^ and recycle previously deposited nucleosomes during genomic transcription^47^. CENP-A loading requires the exchange of placeholder nucleosomes^10,11^, which has been proposed to be mediated by transcription-induced chromatin remodeling^9,22,33^. We therefore hypothesized that Spt6 might also play a role during centromeric transcription and the loading or maintenance of dCENP-A.

**Fig. 2:**
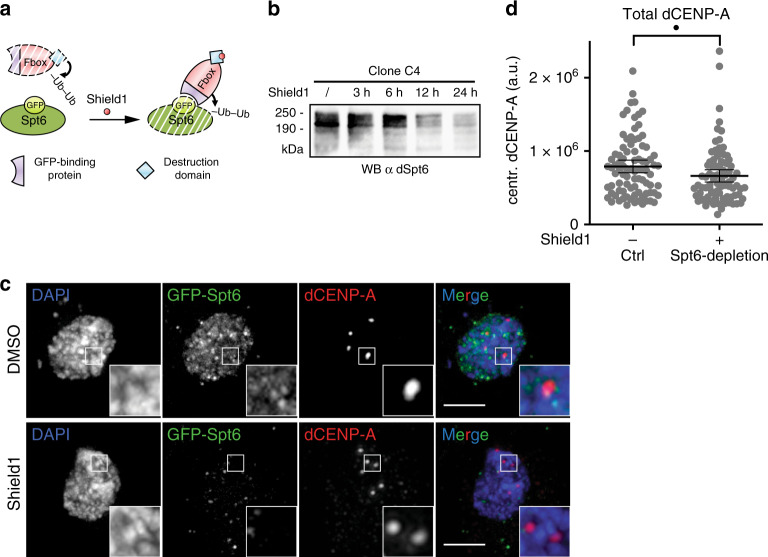
Spt6 depletion results in lower levels of total dCENP-A. **a** Schematic representation of the Shield1-induceable degradation of GFP-Spt6. **b** Western blot analysis demonstrating the depletion of GFP-tagged Spt6 following the addition of Shield1. *N* = 3 independent experiments. **c** Maximum intensity projection of representative cells for the analysis in (**d**). Cells treated for 21 h with DMSO (upper panel) or Shield1 (lower panel) are shown. Boxes indicate the 3× enlarged inset. Scale bar represents 3 µm. **d** Quantification of total centromeric dCENP-A levels in Spt6 WT or depleted cells (17% reduction). *N* = 4 independent experiments; *n*_WT_ = 87, *n*_depl_ = 85 cells. Data are represented as scatter plots with mean and 95% confidence interval (CI). Statistical significance: single dot *P* = 0.0157 (unpaired, two-tailed Mann–Whitney test). Source data are provided as a Source Data file.

As loading of dCENP-A is coupled to progression through the cell cycle, the depletion of Spt6 via RNAi is not suitable to assess potential changes in dCENP-A deposition, especially as the cell cycle block already occurs with Spt6 levels only being reduced to roughly 50% (Supplementary Fig. 2a). To overcome this problem, we used the CRISPR/Cas9 technique to GFP-tag endogenous Spt6 between the second and third exon, inspired by the fully functional GFP-Spt6 protein produced in a GFP-TRAP study (Supplementary Fig. 3a)^52^ and combined it with a system targeting GFP for degradation, called deGradFP^53^. Western blot analysis confirmed that the GFP-fusion protein is the only form of Spt6 present in our stable S2 cell line (Clone C4, Supplementary Fig. 3b). Rapid, inducible degradation of GFP-Spt6 was achieved by adapting the deGradFP system to respond to the small molecule Shield1^54^. Accordingly, we modified the original F-box-construct through addition of a degron domain (FKBP-L106P) that results in constant degradation of the fusion protein. Addition of Shield1 stabilizes FKBP-F-box-GFP Binding Protein, which then initiates the degradation of GFP-Spt6 (Fig. 2a). Indeed, we found that Shield1 induced rapid degradation of Spt6 within 12–24 h as judged by IF of fixed cells and western blot of total protein extracts (Fig. 2b, c). Interestingly, Shield1 treatment for 21 h (<one generation time of 24 h) led to a reduction of total dCENP-A at centromeres in Spt6-depleted cells (Fig. 2c, d), suggesting either impaired loading of new dCENP-A or increased loss of old dCENP-A.

### Spt6 prevents transcription-coupled loss of old dCENP-A

**Fig. 3:**
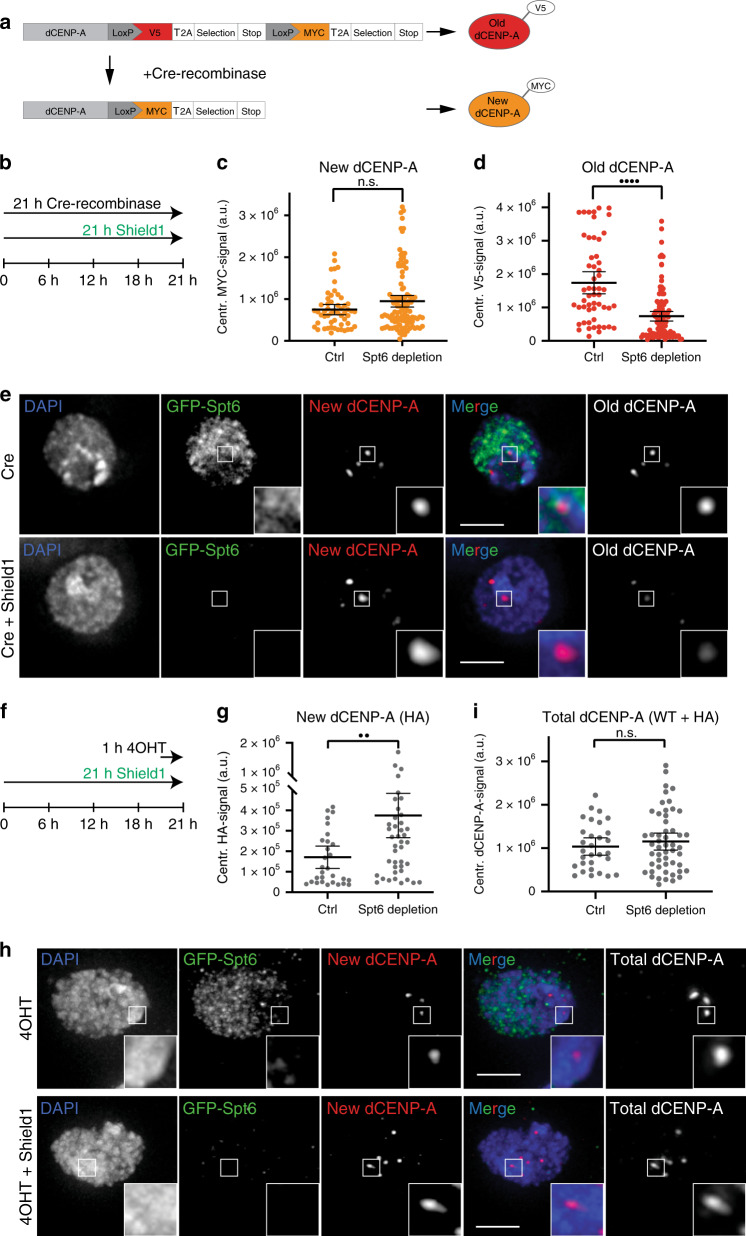
Parental dCENP-A is lost in Spt6-depleted cells. **a** Scheme displaying the RITE system used to distinguish old and new protein simultaneously (adapted from Verzijlbergen et al.^55^). **b** Experimental setup used in (**c**–**e**). Quantification of the centromeric incorporation of new dCENP-A^MYC^ (**c**) or remaining levels of old dCENP-A^V5^ (**d**) at centromeres under Spt6 depletion conditions (58% reduction). *N* = 3 independent experiments; *n*_WT_ = 54, *n*_depl_ = 107 cells. Statistical significance: quadruple dots *P* < 0.0001; n.s. not significant (unpaired, two-tailed Mann–Whitney test). **e** Maximum intensity projection of representative cells measured in **c**, **d**. Cells treated with Cre recombinase (upper panel) or Cre recombinase plus Shield1 (lower panel) are shown. Boxes indicate the 4× enlarged inset. Scale bar represents 3 µm. **f** Experimental setup used in (**g**–**i**). **g** Quantification of new centromeric dCENP-A^HA^ in fixed cells following 4OHT mediated release of TI-dCENP-A^HA^ and depletion of Spt6 through Shield1 treatment. *N* = 3 independent experiments, *n*_WT_ = 30, *n*_depl_ = 47 cells. Data are represented as scatter plots with mean and 95% CI. **h** Maximum intensity projection of representative cells measured in **g**, **i**. 4OHT treated (upper panel) and 4OHT plus Shield1 treated cells (lower panel) are shown. Boxes indicate the 4× enlarged inset. Scale bar represents 3 µm. **i** Quantification of total centromeric dCENP-A in fixed cells following 4OHT mediated release of TI-dCENP-A^HA^ and depletion of Spt6 through Shield1 treatment. *N* = 3 independent experiments, *n*_WT_ = 30, *n*_depl_ = 52 cells. All data plots are represented as scatter plots with mean and 95% CI. Statistical significance: double dots *P* = 0.0025; n.s. not significant (unpaired, two-tailed Mann–Whitney test). Source data are provided as a Source Data file.

To distinguish between a defect in loading versus impaired maintenance of dCENP-A, we established the Recombination Induced Tag Exchange (RITE)-technique^55^ in *Drosophila* S2 cells. This technique allows simultaneous tracking of both new (MYC-tagged) and old proteins (V5-tagged) via a Cre recombinase mediated epitope-tag switch (Fig. 3a). Cells treated with *Cre* recombinase stop transcription of the old and simultaneously start transcription of the new tagged construct. dCENP-A dynamics were assessed after a 21 h treatment with Cre recombinase in the presence or absence of GFP-Spt6 (Fig. 3b). We found that levels of newly loaded dCENP-A^MYC^ were not significantly altered between both treatment conditions, although they showed a tendency toward increased loading in Spt6-depleted cells (Fig. 3c, e). In contrast, old dCENP-A^V5^ was significantly depleted from centromeres in cells where Spt6 was degraded (Fig. 3d, e).

Interestingly, the loss of old dCENP-A^V5^ was more pronounced than the reduction observed for total dCENP-A levels in Spt6-depleted cells (58% vs. 17% reduction; compare Figs. 3d and 2d), suggesting a partial compensation by increased incorporation of new dCENP-A. However, the RITE system did not reveal a significant increase in new CENP-A^MYC^, likely because not enough new protein was produced during the course of the experiment to fully compensate for the loss of old dCENP-A^V5^ (Supplementary Fig. 3c). To test this hypothesis, we combined Spt6 degradation with a previously established tamoxifen-inducible HA-tagged dCENP-A (TI-dCENP-A^HA^) loading system^9^. In this system, TI-dCENP-A^HA^ is constitutively produced but cannot participate in dCENP-A loading, as it is sequestered away in the cytoplasm due to an interaction with Hsp90. Only upon treatment with 4-hydroxytamoxifen (4OHT) is TI-dCENP-A^HA^ released and a large pool of preproduced new dCENP-A proteins becomes instantaneously available for incorporation into centromeric chromatin. Indeed, when combined with simultaneous depletion of Spt6 using our inducible deGradFP system (Fig. 3f), we observed a clear increase in incorporation of new dCENP-A compared with cells with wild-type levels of Spt6 (Fig. 3g, h). Moreover, the presence of endogenous dCENP-A combined with additional TI-dCENP-A^HA^ completely equalized total dCENP-A levels between cells with and without Spt6 depletion, in contrast to the reduction previously observed following Spt6 degradation (compare Figs. 2d and 3i). In support of this interpretation, western blotting of nuclear extracts for dCENP-A revealed that overall protein levels of the transgenic dCENP-A^V5^ constructs in the RITE system are much lower than TI-dCENP-A^HA^ provided by the tamoxifen system (Supplementary Fig 3c, d). In line with our microscopy analysis, we also observed a reduction in total endogenous dCENP-A levels upon Spt6 (Fig. 2d). However, it should be noted that western blots are not well suited to reflect the specific centromeric contribution of CENP-A, as the majority of CENP-A has been found to reside in non-centromeric chromatin (up to 66% in human cells^56^).

### Recombinant Spt6_199–338_ binds dCENP-A and H3 directly

**Fig. 4:**
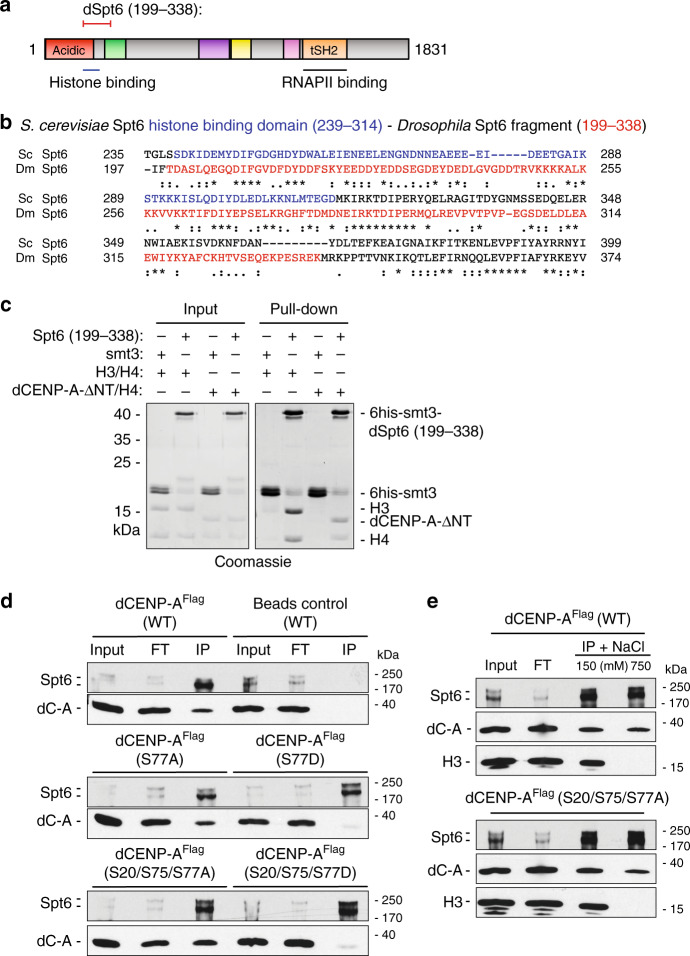
dCENP-A binds directly to Spt6 and is affected by mutating phosphoresidues. **a** *Drosophila* Spt6 domain organization based on Pfam^73^: acidic (red), Helix-turn-Helix (green), YqgF/RNaseH-like domain (purple), Helix-hairpin-Helix (yellow), S1 RNA-binding domain (magenta), tandem SH2 (orange). Corresponding histone and RNAPII-binding domains based on *S. cerevisiae*^39,63^. **b** Alignment of the histone-binding domain of *S. cerevisiae* Spt6 (residues 239–314; blue) based on McDonald et al.^39^ with the bacterially expressed fragment of *Drosophila* Spt6 (199–338; red). Alignments were performed on Uniprot using the Clustal Omega program^74^. Asterisk indicates fully conserved residues, colon indicates strongly similar residues, and period indicates weakly similar residues. **c** Pull-down experiments of purified recombinant 6his-smt3-Spt6 (199–338) or 6his-smt3 as a negative control with recombinant H3/H4 or dCENP-A-ΔNT (101–225) are shown on a Coomassie-stained SDS-PAGE. *N* = 3 independent experiments. **d** Western blot (α FLAG, α Spt6) showing co-IPs of Spt6 with WT dCENP-A^FLAG^ and dCENP-A^FLAG^ bearing phosphorylation-abolishing (S to A) or phosphomimetic (S to D) mutations at phosphorylation sites S20, S75 and S77. *N* = 5 independent experiments. WT wildtype, FT flowthrough; IP immunoprecipitate. dC-A dCENP-A. **e** Western blot (α FLAG, α Spt6, α H3) showing co-IPs of endogenous Spt6 (two bands) with dCENP-A^FLAG^ (top) or dCENP-A^FLAG^ (S20/75/77A) (bottom) and H3 exposed to low (150 mM) and high (750 mM) salt wash conditions. *N* = 5 independent experiments. Source data are provided as a Source Data file.

Intriguingly, the specific loss of previously deposited dCENP-A during the loading process of new dCENP-A in Spt6-depleted cells suggests that Spt6 binds and reincorporates not only H3^47^, but also dCENP-A/H4 tetramers. An association of Spt6 with dCENP-A can be expected from our previously published mass spectroscopy of dCENP-A interactors^45^. Although the exact binding interface is not known, binding of basic histones to the unstructured highly acidic N-terminus of Spt6 (Fig. 4a, b) has been demonstrated in *Saccharomyces cerevisiae*^39^. To test whether the interaction between Spt6 and dCENP-A is direct, we expressed a N-terminal fragment of *Drosophila* Spt6 (residues 199–338), which encompasses the histone-binding domain of yeast Spt6 (residues 239–314) based on amino acid sequence alignments (Fig. 4b). Recombinant Spt6 (199–338) was expressed and purified from bacteria and pull-down experiments revealed direct binding of Spt6 to both recombinant H3/H4 and dCENP-A-ΔNterm (101–255)/H4 tetramers, but not to the epitope tag alone control (6his-smt3) (Fig. 4c). Less H4 was pulled down than H3 or dCENP-A-ΔNterm indicating a potential binding of Spt6 to H3 or dCENP-A alone. As we could only produce soluble dCENP-A without its N-terminal tail in bacteria, we further confirmed that endogenous Spt6 is able to co-immunoprecipitate (IP) with full length dCENP-A^FLAG^ using *Drosophila* S2 cell extracts (Fig. 4d).

### Mutating dCENP-A phosphoresidues affect Spt6 binding

It has been shown that upregulated centromeric transcription in stressed and senescent murine cells can induce removal of CENP-A from centromeres^36^. This was dependent on the DNA damage response effector Ataxia-telangiectasia-mutated kinase and a phosphorylatable S30 residue on CENP-A, suggesting that phosphorylation events act in conjunction with transcription to remove centromeric CENP-A. As Spt6 prevents transcription-coupled loss of nucleosomes in gene bodies^47^, this prompted us to generate cell lines that express Flag-tagged dCENP-A bearing mutations at three previously reported phosphorylation sites of its N-terminal tail (S20, S75, and S77^57^). The respective serines were mutated to either phosphomimetic aspartates or to non-phosphorylatable alanines. In line with a potential negative effect on dCENP-A retention through phosphorylation, a single (S77D) or triple (S20/S75/S77D) substitution to aspartate almost completely abolished co-immunoprecipitation of dCENP-A with Spt6 (Fig. 4d). In contrast, the serine-to-alanine mutation displayed similar (S77A) or modestly increased (S20/S75/S77A) association with Spt6 as compared with wild-type dCENP-A. To test whether Spt6 shows different interaction specificity for H3 and dCENP-A, we exposed the co-IPs to high salt (750 mM) washes. While high salt treatment abolished the association of Spt6 with canonical H3, a clearly detectable population of wild-type dCENP-A^FLAG^ or the non-phosphorylatable triple alanine dCENP-A mutant (S20/S75/S77A) remained bound to Spt6 (Fig. 4e).

### Phosphorylation affects retention of centromeric dCENP-A

**Fig. 5:**
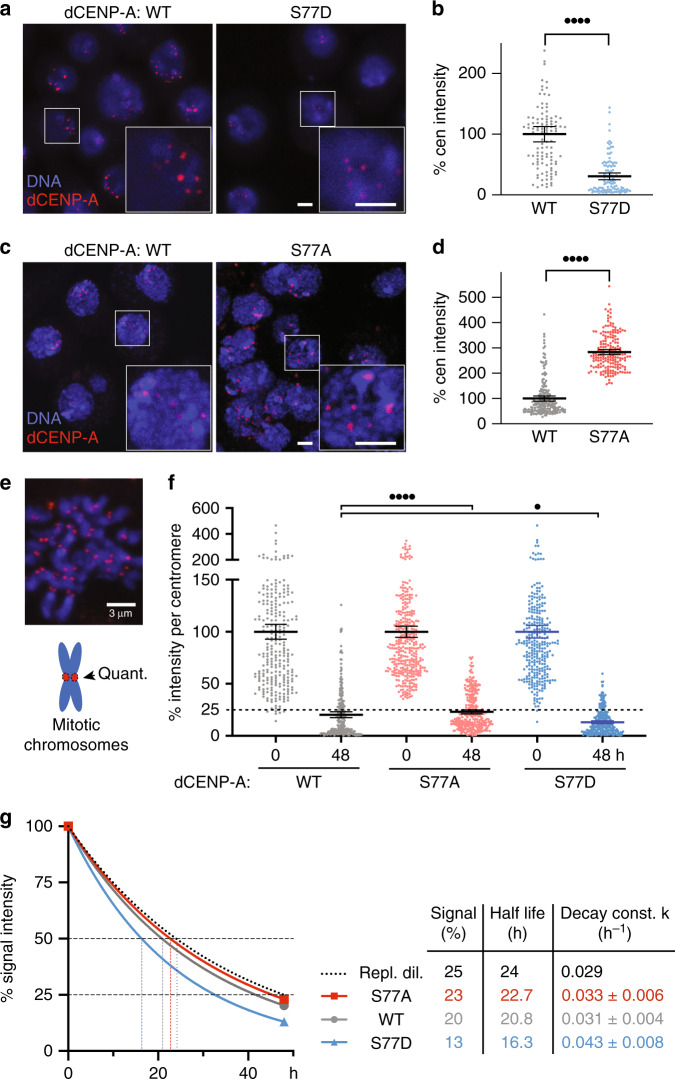
dCENP-A abundance at the centromere is affected by phosphorylation. **a** Stably transfected cells with SNAP-tagged wildtype (WT) or S77D mutant dCENP-A were visualized by staining with TMR Star. Boxes indicate the 2.5 times enlarged inset. **b** Quantification of centromeric signal intensities of wildtype (*n* = 196) and S77A CENP-A (*n* = 182 centromeres). Representative images (**a**) and quantification (**b**) of one out of *N* = 3 independent experiments are shown. **c** SNAP-tagged wildtype and S77A-mutant dCENP-A staining by TMR Star. **d** Quantification of centromeric signal intensities of wildtype (*n* = 110) and S77D CENP-A (*n* = 112). Representative images (**c**) and quantification (**d**) of one out of *N* = 5 independent experiments. Values are normalized relative to the wildtype mean (set to 100%). Data are represented as scatter plots with mean and 95% CI. Statistical significance: quadruple dots *P* < 0.0001 (unpaired, two-tailed Mann–Whitney test). **e** Example image of a mitotic chromosome spread (wildtype) and cartoon illustrating analysis shown in (**f**). **f** Quantification of total TMR signal intensities per centromere (representative images shown in Supplementary Fig. 4), *n*_WT 0|48h_ = 284|251, *n*_S77A 0|48h_ = 368|305, *n*_S77D 0|48_ = 315|261 is shown for one out of *N* = 3 independent experiments. Mean values for the 0 h time point was set to 100% for each cell line. The dashed line marks 25%, the signal intensity expected for dCENP-A undergoing only replicative dilution after two cell generations (48 h). Data are represented as scatter plots with mean and 95% CI. Statistical significance: quadruple dots *P* < 0.0001, single dots *P* = 0.0221 (unpaired, two-tailed Mann–Whitney test). **g** Non-linear regression curves illustrating the decrease of centromeric CENP-A signal over 48 h. Half-life and decay constant *k* (± upper and lower 95% CI) were calculated using the one-phase-decay function of Prism 8.4.0. Source data are provided as a Source Data file.

Next, we tested whether the altered interactions between Spt6 and dCENP-A-mutants also correlate with changed centromeric levels of dCENP-A. We quantified the steady-state level of SNAP-tagged-dCENP-A at centromeres in stably transfected S2 cells that express wild-type or mutant constructs (S77D or S77A; Fig. 5a–d). Consistent with the co-IP results, we find that centromeric dCENP-A-S77D intensities were strongly decreased (≈3-fold vs. WT; Fig. 5a, b). Surprisingly, we also observed a significantly higher abundance of dCENP-A-S77A in centromeres as compared with wild-type dCENP-A (≈2.8-fold, Fig. 5c, d). An accumulation of dCENP-A-S77A is unexpected, if endogenous CENP-A is only subjected to replicative dilution^12^ but otherwise 100% stable.

We therefore investigated retention of dCENP-A in more detail using a SNAP-based fluorescent pulse-chase approach to follow the decline of old wildtype, S77A or S77D dCENP-A over 2 cell generations (48 h) at individual mitotic centromeres (Fig. 5e, f, g Supplementary Fig. 4). Interestingly, we uncovered a low level of wildtype dCENP-A loss after two cycles in addition to replicative dilution (20% instead of the expected 25% of the original intensity), in agreement with recent findings that challenge the high intrinsic stability of CENP-A^13^. In contrast, the retained levels of the S77A mutant are closer to perfect inheritance (23%), while, as expected, the remaining amounts of the phosphomimetic mutant are much lower (13%). This difference is further revealed by calculating the half life and decay rate constant for each mutant after 48 h (Fig. 5g), providing an explanation for how the S77A mutant can accumulate relative to wild-type dCENP-A.

### Depletion of human SPT6 interferes with CENP-A maintenance

**Fig. 6:**
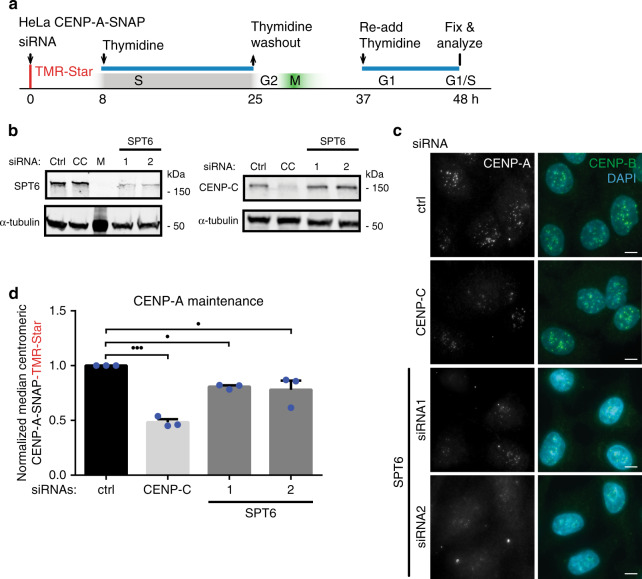
Depletion of human SPT6 leads to the loss of CENP-A maintenance. **a** Experimental setup. HeLa cells expressing SNAP-tagged CENP-A were treated with TMR-star to detect previously incorporated CENP-A and siRNA-treated to deplete proteins indicated in (**b**, **c**). Cells were then synchronized in S phase by a thymidine block and released. Cells were allowed transit through G1 phase and were collected at the next G1/S boundary by re-addition of thymine. **b** Cells were treated with indicated siRNAs for 48 h and extracts were processed for immunoblotting and probed with indicated antibodies. CC CENP-C, M Marker. *N* = 3 independent experiments. **c** Representative images of siRNA-treated cells, 48 h after TMR pulse labeling and mRNA depletion. Cells were counter stained with DAPI and anti-CENP-B antibodies to label DNA and centromeres, respectively. Scale bar represents 10 μm. **d** Quantification of experiments shown in (**a**, **c**). Mean and ± SEM of *N* = 3 independent experiments is shown normalized to median control siRNA (ctrl). Images are quantified with CrAQ2. *n*_Crtl_ = 3989, *n*_CC_ = 4097, *n*_Spt6_1_ = 3635, *n*_Spt6_1_ = 3559 centromeres. Statistical significance: triple dots *P*_CC_ = 0.0001, single dots P_1_ = 0,0374, single dots P_2_ = 0.019. (One-way ANOVA, Dunnett’s multiple comparisons test). Source data are provided as a Source Data file.

To determine whether our observations are generalizable across species, we depleted the highly conserved SPT6 protein in human HeLa cells. We measured the rate of CENP-A retention across the cell cycle using SNAP-based fluorescent pulse labeling that specifically tracks ancestral chromatin-bound CENP-A^12,58^. As in *Drosophila* S2 cells, strong depletion of SPT6 results in secondary phenotypes including a cell cycle arrest which indirectly interferes with CENP-A loading by blocking passage through M- and G1 phase. To prevent this, we partially depleted SPT6 (Fig. 6b) using specific siRNAs and controlled the cell cycle speed by a Thymidine arrest and release protocol to allow SPT6-depleted cells to complete a cell cycle (Fig. 6a). We ensured cells reached G1 phase based on the lack of Cyclin B staining (Supplemental Fig. 5a, b). Under these conditions we observe a reproducible 20% reduction in CENP-A maintenance in a single cell cycle. Two different siRNAs resulted in similar effects, indicating CENP-A loss is due to on target SPT6 depletion (Fig. 6b, c). We expected this effect to be modest as expression of SPT6 is by necessity hypomorphic. CENP-C, a previously identified CENP-A maintenance factor, was used as control^14^. Although further investigation will be required to exclude the possibility of unspecific effects on CENP-A stability, SPT6 depletion did not affect CENP-C levels indicating that the effect of SPT6 depletion on CENP-A is not an indirect consequence of CENP-C loss (Fig. 6b, right panel). Unlike what has been observed for fission yeast^22^, we did not detect CENP-A mislocalisation after Spt6 depletion in either *Drosophila* or human cells. In summary, these data support a conserved role for SPT6 in CENP-A maintenance in human cells.

## Discussion

The CENP-A nucleosome is considered to be the key epigenetic mark for centromere identity in most organisms. Accordingly, CENP-A and epigenetic marks in general should meet three requirements: (1) Template its own deposition, (2) be replenished in a cell cycle-controlled manner to counteract dilution by half in each S-phase and (3) be stably transmitted to the next cell generation^59^.

New dCENP-A can be targeted to sites of previous CENP-A deposition by its chaperone CAL1, which is recruited to centromeres by dCENP-C^60,61^. Loading of new CENP-A is restricted to mitosis and G1^5–8^ and serves primarily to replenish CENP-A containing nucleosomes that became diluted by half during the preceding S-phase. During DNA replication the MCM2-7 replicative helicase along with other histone chaperones like HJURP, are instrumental for the stable transmission of parental CENP-A during S-phase^19–21^.

**Fig. 7:**
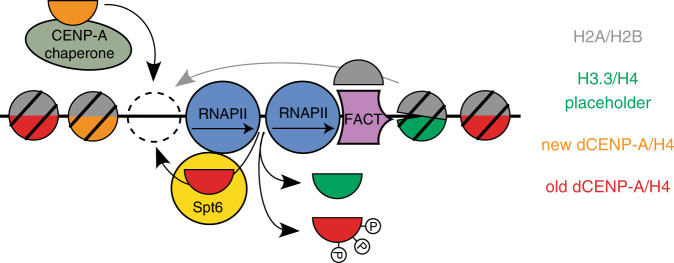
Model showing histone dynamics during dCENP-A loading. Both old CENP-A and placeholder H3 nucleosomes can be evicted during the transcription-induced remodeling of centromeric chromatin. Spt6 functions to retain old CENP-A nucleosomes. Phosphorylation of CENP-A interferes with Spt6-mediated recycling and maintenance.

We have recently shown in *Drosophila* S2 cells that transcription at the centromere is required for stable nucleosome incorporation of new dCENP-A^9^. This finding could be explained by a model in which transcription-mediated chromatin remodeling is re-purposed to evict placeholder H3 nucleosomes to make room for deposition of new dCENP-A. However, the induction of nucleosome eviction during CENP-A loading also bears the danger of losing previously incorporated CENP-A (Supplementary Fig. 6). Here we report the identification of the transcription elongation factor and histone chaperone Spt6 as a new CENP-A maintenance factor, which safeguards previously deposited CENP-A during centromeric transcription (Fig. 7).

We find that *Drosophila* Spt6 localizes to centromeres during mitosis and G1 (Fig. 1 and Supplementary Fig. 1), coinciding with the time window when transcription and dCENP-A loading occurs^6–9^. The SH2 domain enables Spt6 to interact directly with RNAPII^48,62,63^ and it is therefore likely that recruitment of Spt6 to centromeres is a direct consequence of centromeric transcription. Because Spt6 prevents transcription-coupled loss of posttranslationally modified nucleosomes in gene bodies^38,47^, we tested whether Spt6 might act to maintain dCENP-A at the centromere. Indeed, when we depleted Spt6 in *Drosophila* or human cells, we observed the specific loss of old CENP-A after passage through mitosis into G1 phase (Figs. 3 and 6). This observation suggests that ongoing transcription evicts nucleosomes at centromeres and that Spt6 serves a conserved role to recycle CENP-A/H4 tetramers expelled by closely spaced polymerase complexes^17^. A key point of this model is the transcription-mediated creation of nucleosomal gaps as a prerequisite for full incorporation of new dCENP-A (Fig. 7^9^). Consequently, the additional loss of nucleosomes in Spt6-depleted cells should create more opportunities to load new dCENP-A. Indeed, when we used an experimental system that provides elevated levels of transgenic, ready-made dCENP-A (TI-dCENP-A^HA^), we observed a clear increase in loading (Supplementary Fig. 3d, Fig. 3g, h). This is further supported by the fact that the loss of total centromeric dCENP-A in Spt6-depleted cells is completely compensated under these conditions (compare Figs. 2d and 3i).

We currently do not know if the mitotic defects observed upon Spt6 depletion by RNAi are a direct or indirect consequence of Spt6 removal (Supplementary Fig. 2b–d). As cells can tolerate very low CENP-A levels at the centromere down to 10%^64^, the 2-day depletion of Spt6 likely leaves sufficient dCENP-A for centromere function. Despite this, chromosome segregation might be compromised due to the specific loss of old nucleosomes with specific PTMs. PTMs relevant for centromere function have been identified on CENP-A and shown to affect CENP-A stability and correct mitotic progression^65,66^. Moreover, methylation of lysine 20 on the associated H4 plays an essential role for kinetochore formation^67^. Likewise, in addition to CENP-A nucleosomes, centromeres contain canonical H3 nucleosomes with a specific set of posttranslational modifications that might need to be retained^24,31^. We therefore postulate that Spt6 should be able to distinguish between placeholder nucleosomes that need to be removed and epigenetically marked nucleosomes that should be kept. As previously demonstrated for H3/H4 in budding yeast^39^, we observe direct binding of a bacterially expressed N-terminal fragment of Spt6 (199–338) with both H3/H4 and dCENP-AΔNT/H4 tetramers (Fig. 4b). In addition, full length dCENP-A^FLAG^ and H3 co-IP with endogenous Spt6 from S2 cell extracts with comparable efficiency (Fig. 4e).

Interestingly, CENP-A is phosphorylated in various organisms including flies and humans^57,68,69^ and phosphorylation events have been linked to transcription-induced loss of centromeric CENP-A nucleosomes in mouse cells^36^. To test whether phosphorylation of dCENP-A affects its maintenance, we mutated three previously identified phosphorylation sites in the N-terminal tail of dCENP-A (S20, S75 and S77^57^). Indeed, we found that dCENP-A mutants carrying the phosphomimetic residue aspartate showed significantly reduced binding to Spt6, while the opposite was observed for the respective non-phosphorylatable alanine mutants (Fig. 4d). Furthermore, wild-type or non-phosphorylatable mutants of dCENP-A bound robustly to Spt6 when exposed to high salt washes while canonical H3 binding was abolished (Fig. 4e). This difference hints toward a mechanism how Spt6 distinguishes between the two histone H3-variants and allows selective retention of CENP-A, while placeholder nucleosomes are exchanged. Consistent with the observations described above, a pulse-chase experiment to follow the decline of old dCENP-A during cell division showed higher loss rate for the phosphomimetic dCENP-A construct. Interestingly, dCENP-A wild-type displayed less than perfect inheritance after two cell cycles (<25% expected for replicative dilution). In contrast S77A was on average more and S77D less stable than wild type (Fig. 5f), likely accounting for the accumulation of the non-phosphorylatable dCENP-A mutant at centromeres over time (Fig. 5c).

Taken together, we propose that the transcription-mediated eviction of centromeric nucleosomes affects both placeholder H3 and previously deposited CENP-A nucleosomes. However, loss of the centromeric mark is prevented by specific recycling of CENP-A through Spt6, potentially involving phospho-regulation of the CENP-A/Spt6 interaction (Fig. 7). We conclude that Spt6 acts as an important CENP-A maintenance factor and contributes to the long-term stability of the epigenetic centromere mark.

## Methods

### Plasmid constructs

Spt6 was PCR’ed from genomic S2 cell cDNA and cloned into the SpeI/EcoRV sites of a pMT-CID-GFP-hygro vector (primers dSpt6_F1, dSpt6_R2) and a pIB-vector (Invitrogen; primers dSpt6_F1, dSpt6_R-AscI), creating pMT-Spt6-GFP-hygro and pIB-resSpt6-stop. The pMT_FKBP_L106P_Fbox_GFPbinder_hygro_opt plasmid was constructed for the degradFP technique by cloning two gBlock DNA fragments (IDT) for the FKBP_L106P degron (KpnI/SpeI) and the F-box-GFP binder (SpeI/XbaI) into the pMT_V5_hygro. CID-HA-ERT2 (=TI-dCENP-A^HA^)^9^ is subcloned using KpnI/AgeI into pMT_kana_opt. The pRITE_MT_CID_V5_puro_3myc_CD4 plasmid used for RITE was constructed by combining several synthesized gBlock DNA fragments (IDT) and a PCR-fragment containing the CD4 gene into the NotI/PmeI site of a pMT-CID-V5 plasmid (for sequence details, see Supplementary Table 1). The 1kup_resGFPSpt6_stop-plasmid was generated by inserting the PCR’ed genomic region of Spt6 1 kb upstream fragment (primers 1kup_F_BstZ17I, 2kup_R_NheI) into the pIB-Spt6 vector using BstZ17I/NheI. A gBlock containing a synthesized artificial GFP-exon (resGFPSpt6) between the 2nd and 3rd Spt6-exon using HindIII and Eco47III. pET3a_ΔN_CENP-A_101–225_ is described^70^, pET22b H3 and pET22b H4 were a gift from Karolin Luger. The Spt6199-338 fragment was produced as a gBlock (IDT) and used directly in ligation-independent cloning into pET-His6-Sumo TEV (14S Addgene plasmid # 48291), a gift from Scott Gradia. To generate dCENP-A-S77A or -S77D single mutant constructs, primers S77A_F and S77A_R (or S77D_F and S77D_R) were used for site-directed mutagenesis on pMT-SNAP-CENP-A^57^ as template. To generate dCENP-A-S20/75/77A and CENP-A-S20/75/77D triple-mutant constructs, first a pMT-S20A (pMR-S20D) single mutant construct was made with primers S20A_F and S20A_R (S20D_F & S20D_R) as above, the resulting plasmid was used for site-directed mutagenesis of S75 with primers S75A_R and S75A_R (S75D_F & S75D_R) followed by site-directed mutagenesis of S77 with primers B75AM77A_F and S77A_R (B75DM77D_F & B75DM77D_R). For primer and gBlock sequences see Supplementary Table 1.

### Cell culture and generation of cell lines

*Drosophila* S2 Schneider cells were grown at 25 °C in Schneider’s Drosophila medium (SERVA) supplemented with 10% fetal calf serum and antibiotics (0.3 mg/ml penicillin, 0.3 mg/ml streptomycin). Cells were transfected using the XtremeGENE HP transfection reagent (Roche) or Effectene Transfection Reagent (QIAGEN), and stable lines were selected with 100 μg/mL hygromycin B, 2 μg/ml puromycin or 1 mg/ml G418 (kanamycin derivative). Degradation of Spt6 was induced in the CRISPR generated GFP-Spt6 cell line clone C4 stably expressing the FKBP-deGradFP construct by treatment for 21 h with 4 μM Shield1 in 1% DMSO (D8418, SIGMA) final concentration in the cell medium. Clone C4 was stably transfected with the pMT_dCENPA-HA-ERT2-hygro (TI-dCENP-A^HA^) construct or the pRITE_MT_CID_V5_puro_3myc_CD4 plasmid. Clone C4 expressing cytoplasmic-ankered TI-dCENP-A^HA^ was exposed to 10 μM 4-hydroxy-tamoxifen (4OHT; T176, Sigma) for the last hour of a 21-h Shield1 (#632189, Takara) or 1% DMSO control treatment. Clone C4 expressing the RITE construct was induced for recombination by direct addition of 1 μM Cre Recombinase (TAT-Cre, EG-1001, Excellgen) to the cell medium for 21 h in the presence of Shield1 or absence (1% DMSO control).

HeLa CENP-A-SNAP cells were grown at 37 °C, 5% CO_2_ in DMEM cell culture media supplemented with 10% newborn calf serum, 2 mM glutamine, 1 mM sodium pyruvate (SP), 100 U/ml penicillin, and 100 μg/ml streptomycin. The HeLa monoclonal cell lines stably expressing CENP-A-SNAP were a gift from Don W. Cleveland (UCSD) and is the same cell line used for many centromere studies in human cells (clone#72)^5^.

### RNAi

Exponentially growing S2 cells were incubated for 30′ in 1 ml serum-free medium containing 20 µg of dsRNA before 3 ml of serum-containing medium were added. Cells were harvested after 3 (white/Spt6) or 7 (dCENP-A) days. Primers used for dsRNA synthesis: Spt6_F, Spt6_R, dWhite_F, dWhite_R,

RNAi in HeLa cells was performed in a 24-well format with 2.5 pmol of small interfering RNAs final concentration (siRNAs) using Lipofectamine^®^ RNAiMAX Transfection Reagent (Invitrogen) according to the manufacturer’s instructions. All siRNAs were obtained from Silencer^®^ Select Pre-Designed & Validated siRNA (Life Technologies): CENP-C (s2913), SPT6 (s13634, s13635). Neg9 depletion siRNA target was used as a control (ctrl). Following RNAi, HeLa cells were synchronized by addition of 2 mM thymidine to complete medium for 17 h. Cells were released into G2 and through G1 by washing them twice in complete medium before incubating them in complete media containing 24 µM deoxycytidine. Cells were collected by re-addition of Thymidine, as outlined (Fig. 6a).

### Immunofluorescence

Generally, S2 cells were settled for 20′ on polylysine coated cells and fixed for 7′ with 3.7% formaldehyde solution (Sigma) in phosphate-buffered saline (PBS) or in −20 °C methanol. For staining of endogenous Spt6 at centromeres, shorter fixation conditions (3′ in PBS/0.1% Triton (PBS-T) containing 1.85% formaldehyde) were required. Following a wash in PBS-T, samples were blocked with Image-iT^TM^FX signal enhancer (Invitrogen) for at least 30′. Samples were stained at 4 °C overnight in PBS-T containing respective primary antibodies. Unless otherwise noted, all antibodies were used 1:100 diluted: chicken α dCENP-A (H31, 1:20; P. Heun, polyclonal), monoclonal rat α dCENP-A (4F8; E. Kremmer, Helmholtz Zentrum München), rabbit α H3S10p (Abcam, ab5176, polyclonal), mouse α GFP (Clone 496; D. van Essen, monoclonal), mouse α tubulin (Sigma, T5168) and mouse α V5 (Invitrogen, Cat# R960-25, monoclonal). Monoclonal antibodies were raised in the Helmholtz Zentrum München in rats or mice against Spt6 amino acid residues 215–230 DYDDFSKYEEDDYEDD (mouse α dSpt6 (26D12, IgG2b, 1:50), rat α dSpt6 (13D4, IgG1; 1:50)). Secondary polyclonal goat antibodies (Invitrogen) coupled to Alexa 488, Alexa 555 and Alexa 647 fluorophores used (in the same order) were anti-rabbit (A-11070, A-21429, A-21246), anti-mouse (A-11001, A-21422, A-21235), anti-chicken (A-11039, A-21437, A-21449) and anti-rat for Alexa 488 (A-11006) were used at a 1:500 dilution and incubated for 45′ at room temperature. Counterstaining of DNA was performed with 4′,6-diamidino-2-phenylindole (DAPI) (5 µg/ml; 3′).

HeLa cells were fixed for 10′ at room temperature fixation in 500 µl of PBS/4% formaldehyde solution. After aspiration, samples were quenched through addition of 0.1 M Tris, pH 7.5 for 5′. Samples were permeabilized through a 5′ wash in PBS-T and blocked for 30′ in PBS containing 2%FBS, 2% BSA, 0.1% Triton X-100 and 0.04% NaN_3_ At 37 °C. Antibody labeling was performed at 37 °C for 60′ (primary) and 30′ (secondary) respectively. Antibodies against CENP-B (Santa Cruz Biotechnology, sc-22788 Rabbit polyclonal) and cyclin B1 (sc-245; Santa Cruz Biotechnology) were used at dilutions of 1:1000 and 1:50. Fluorescent secondary antibodies Donkey anti-Rabbit FITC (Rockland, 611-702-127) and Donkey anti-mouse 680 (Rockland, 610-744-124) were used at a dilution of 1:200.

### Snap labeling

Stable SNAP-dCENP-A expressing S2 cells were labeled with TMR-Star (New England Biolabs) at 3 µM final concentration for 30′, followed by three washing steps with fresh culture medium for 10′ each. To assess steady-state levels, cells were fixed as described below and subjected to IF or for pulse-chase labeling with further incubation with 10 µM BTP (SNAP-Cell Block; New England Biolabs) for 30′ and three washes with fresh culture medium. Cells were collected for mitotic chromosome spread preparation (see below) at 0 h and 48 h after BTP blocking and washing steps.

SNAP labeling in HeLa cells was performed for 15′ with 2 μM TMR-Star (New England Biolabs) in complete medium for pulse labeling, after which cells were washed twice with PBS and reincubated with complete medium. After an additional 30′, cells were washed once more with PBS and reincubated with complete medium with 2 μM BTP (SNAP-Cell Block; New England Biolabs) for 30′ and again washed with PBS and further treated for analysis, as indicated^12^ (Fig. 6a).

### Mitotic chromosome spreads

A total of 0.2 × 10^5^ cells were arrested for 30′ with 1 μg/ml colcemid. Supernatant after centrifugation (3′, 1000 g) was discarded, and cells were resuspended in 500 μl of 0.5% (wt/vol) sodium citrate. After 10′ incubation, each sample was transferred into a single-chamber cytospin tunnel and spun on a polylysine- coated slide for 10′ at 91.45 g (900 rpm; high-acceleration) in a Shandon Cytospin 4. After the spin, slides were immediately fixed in 3.7% formaldehyde solution (Sigma), then washed twice in PBS followed by two washes in 2× SSC buffer.

### Click-it chemistry

S-phase cells were visualized using the Click-iT^®^ EdU Imaging Kit from Thermo Fisher Scientific following the manufacturer’s instructions (labeling: 15′/10 µM EdU). Global RNA transcription was detected using the Click-iT^®^ RNA Imaging Kit from Thermo Fisher Scientific (labeling: 5′/4 mM EU). Cells were pelleted and resuspended twice in 1 ml of medium to allow unbound EU to diffuse (5′; 1000 g), before cells were settled and fixed as usual. Click-it reaction was performed according to the manufacturer’s instruction.

### Microscopy and image analysis

All images (see exception below) were taken on a DeltaVision Elite Imaging System and were deconvolved using softWoRx Explorer Suite (Applied precision). Images of fixed cells were taken as 50–65z stacks of 0.2 µM increments using a 100× oil immersion objective. Time-lapse imaging was performed with 25z stacks of 0.4 µM increments using a 60× oil immersion objective and a time-lapse of 2′. Quantification of signal intensities was performed using the softWoRx Explorer Suite. Average background of five non-centromeric nuclear measurements was subtracted from measured centromeric signal (dCENP-A, HA, V5 and MYC analysis) and average background of five cytoplasmic measurements was subtracted from five measured nuclear signals (Spt6). Images of SNAP-tagged dCENP-A expressing S2 cells were taken on a Leica TCS SP5 instrument. Image acquisition was performed using a 63× oil objective with a pixel size of 48.1 nm and by collecting 0.13 µm z-sections spanning the entire nuclei. 3D images were reconstructed and analyzed by Imaris v9.3. Centromeric TMR signals were quantified by measuring the cumulative intensity of each spot. For better comparability, all intensity signals were expressed as % relative to the 100% mean of the 0 h time point in each cell line. To calculate the stability of the different CENP-A mutant and wildt-ype proteins at the centromere over time, all values were normalized to the respective 0 h samples. dCENP-A half-life and decay constant *k* were calculated by determining average dCENP-A fluorescence intensities at 0 and 48 h (two generations). A non-linear fit regression curve was produced using the one-phase-decay function in GraphPad Prism 8.4.0 based on *F* = e^−kt^, where *F* is fluorescent signal intensity and *t* is number of divisions, with a constrained *F*_0_ = 100 and Plateau = 0. Half-life = ln(2)/*k* (Fig. 5g). HeLa cells were imaged on a DeltaVision Core system (Applied Precision) and centromeres were quantified with ImageJ with the CRaQ plugin^12,58^. For imaging a 60× oil objective was used with NA 1.42, working distance 0.15.

### Whole cell and nuclear extracts

All steps were carried out on ice/at 4 °C and all used buffers contained in addition Protease inhibitor cocktail tablets (cOmplete Tablets; Roche) and 0.5 mM PMSF. Cells were washed twice with PBS before lysis. For whole cell extracts pelleted cells were resuspended in buffer L (50 mM Tris-HCl, 150 mM NaCl, 1 mM EDTA, 1% Triton X-100, 1 mM MgCl_2_,) and sonicated 10× with an interval of 30″ on medium setting (Bioruptor300; Diagenode). Protein concentrations were measured using Quick Start^TM^ Bradford 1× Dye Reagent from BIO RAD according to the manufacturers manual and equal amounts loaded for each lane. For nuclear extracts, pelleted cells were incubated for 10′ in lysis buffer (10 mM Hepes-KOH, 1.5 mM MgCl2, 10 mM KCl, 0.5 mM DTT). Nuclei were pelleted and washed once with lysis buffer, resuspended in extraction buffer (20 mM Hepes, 25% glycerol, 420 mM NaCl, 1.5 mM MgCl2, 0.2 mM EDTA, and 0.5 mM DTT) plus Benzonase (Novagen; 100 U/ml) and incubated on ice for 20′. Supernatant after centrifugation served as the nuclear extract.

### Western blot analysis

Drosophila S2 cell samples were boiled for 10′ in loading buffer separated on 6% (Spt6) or 10% (Co-IPs) SDS-PAGE gels and processed for western blotting using mouse α GFP (1:2000, Clone 71; D. van Essen), monoclonal mouse α Spt6 (25C6, Helmholtz Zentrum München, IgG2b 1:500) was raised against Spt6 amino acid residues 215–230 DYDDFSKYEEDDYEDD, rabbit α CENP-A (1:5000, ab10887, Abcam), mAB α Flag (Clone M2; 1:10000, F1804, Sigma-Aldrich), rabbit α H3 (1:10000, ab1791, Abcam) and mouse α tubulin AA4.3 (1:1000; DSHB). Secondary polyclonal Goat antibodies (Sigma) coupled to horseradish peroxidase Rabbit IgG HRP Linked Whole Ab, (NA934) and Mouse IgG HRP Linked Whole Ab (GE Healthcare, NA931) were used at 1:10000. HeLa cells were boiled in 2× Laemmli sample buffer and whole cell extracts were separated by SDS-PAGE and transferred onto nitrocellulose membranes using a Trans Blot System (BioRad). Antibodies used were SPT6 (Abcam, ab32820 rabbit polyclonal; 1:500), CENP-C (Gift from D. Cleveland UCSD, made in house from Covance clone #3024, rabbit polyclonal; 1:10000) and α-tubulin (Sigma, T9026 mouse monoclonal; 1:1000). Secondary antibodies used were Donkey anti-Rabbit 800 (Li-Cor, 926–32211) and Donkey anti-mouse 680 (Rockland, 610-744-124; both at 1:10000). Blots were visualized using Image Lab 6.1 (BIO RAD).

### Co-immunoprecipitation

All steps were carried out on ice/at 4 °C, all used buffers contained in addition protease inhibitor cocktail tablets (cOmplete Tablets; Roche) and 0.5 mM PMSF. Mouse anti-Spt6 antibody (25C6, IgG2b) was coupled to Protein A-Sepharose CL-4B (GE Healthcare) beads and stored at 4 °C until actual pulldown. For nuclei purification, 50–100 × 10^6^ cells were pelleted (10′; 1000 × *g*) and resuspended in 5 ml of nuclear buffer A (85 mM KCL, 5.5% sucrose, 10 mM Tris-HCl pH 7.5, 0.2 mM EDTA, 0.5 mM spermidine). Five mililiter of nuclear buffer B (as A but containing 0.5% NP-40) was added and samples incubated for 3′ on ice. After centrifugation of nuclei (10′; 2000 × *g*), supernatant was discarded, the pellet was washed twice with hypotonic buffer (20 mM HEPES-KOH pH7.9, 20 mM NaCl, 5 mM MgCl_2_, 10 mM imidazole and 0.5 mM β-mercaptoethanol) and resuspended in 300 µl of hypotonic buffer containing 0.5% NP-40 (Sigma-Aldrich) and 200 U Benzonase (Novagen) and incubated with rotation for 1 h. NaCl was added to a final concentration of 300 mM and samples were rotated for 1 h before salt concentration was brought to 150 mM through dilution with hypotonic buffer containing 0.5% NP-40 (Sigma-Aldrich). Ten percent of the supernatant after centrifugation (15′; 15000 × *g*) served as input and the remaining sample was either split into two or three equal samples. Antibody-bound sepharose beads were added to each sample and rotated overnight. Ten percent of the unbound supernatant served as flowthrough and beads were washed for 1 h with hypotonic buffer containing 0.5% NP-40 and either 150 mM or 750 mM NaCl (Fig. 4e). For Fig. 4d, washes were performed with 150 mM salt buffer. Ten percent of each supernatant served as the wash sample, beads were washed three additional times for 2′ with buffer containing the respective salt concentration before bound proteins were eluted by addition of 2× Laemmli sample buffer to obtain the IP sample.

### Expression of recombinant proteins and pull-down assay

6his-smt3-Spt6 (199–338) and 6his-smt3 were recombinantly expressed in bacteria. Histones H3, H4, and CENP-A-ΔNT (101–225) were purified. Histones H3, H4, and CENP-A-ΔNT (101–225) were expressed in *Escherichia coli* solubilized from inclusion bodies and purified by sequential anion and cation exchange chromatography. H3/H4 and CENP-A-ΔNT (101–225)/H4 histone tetramers were assembled by dialysis into refolding buffer (10 mM Tris pH 8, 2 M NaCl, 1 mM EDTA, 5 mM ß-mercaptoethanol) and purified by size exclusion chromatography on a Superdex 200 column^71^. 6his-smt3-Spt6 (199–338) and 6his-smt3 were incubated with dCENP-AΔNT(101–225) or H3/H4 in Pull-Down Buffer containing 20 mM Tris-HCl pH 8, 0.5 mM β-Mercaptoethanol, 10 mM imidazole, 0.1% Triton X-100, 5% glycerol and a final concentration of 250 mM NaCl on an overhead rotator for 1 h at 4 °C. Afterward, input was taken and 20 µl HIS-Select^®^ HF Nickel Affinity Gel (Sigma) pre-equilibrated with Pull-Down Buffer was added to each sample and incubated on an overhead rotator for 1 h at 4 °C. Beads were washed 5× with 1 ml Pull-Down Buffer, bound proteins eluted with Laemmli Buffer at 95 °C for 5′ and analyzed on 15% SDS-PAGE gel stained with InstantBlue™ (Expedeon).

### CRISPR/Cas9

Guide RNA sequences were selected using the flycrispr-tool http://targetfinder.flycrispr.neuro.brown.edu/; Spt6-sgRNA5:TGACGACTTCTCAAAGTACGAGG; Spt6-sgRNA9: GGTTACGATTCCGATGGCGTCGG. Corresponding forward and complementary reverse primers were hybridized and cloned into pAc-sgRNA-Cas9^72^, a gift from Ji-Long Liu (Addgene plasmid # 49330; http://n2t.net/addgene:49330; RRID:Addgene_49330) using SapI creating pAc-Spt6-sgRNA5-Cas9 and pAc-Spt6-sgRNA9-Cas9. The U6 sgRNA region containing sgRNA5 and 9 was amplified using dU6_2_sgRNA_F and dU6_2_3 sgRNA_R primers and the PCR product was transfected into S2 cells along with a plasmid pIB_Cas9_CD4_Blast and the plasmid providing the GFP-tagged DNA repair template (1kup_resGFPSpt6_stop). After 2 days, transfected cells were selected for 4 days with blasticidin. GFP-positive cell clones were picked under the microscope and amplified to produce clone C4 with all alleles tagged.

### Flow cytometry cell cycle analysis

Cells were pelleted in a FACS tube (7′, 1000 × *g*) and fixed at 4 °C overnight in 70% ethanol. Fixed cells were stained for 1 h in the dark (50 µg/ml propidium iodide, 100 µg/ml RNase in PBS) and directly subjected to analysis on a BD FACScalibur Flow Cytometer using a gate for single cells with BD FACSDiva 8.0.1 (gating strategy is provided as Supplementary Fig. 7) and analyzed by FlowJo 10.1.

### Reporting summary

Further information on research design is available in the Nature Research Reporting Summary linked to this article.

## Supplementary information

Supplementary Information

Peer Review File

Supplementary Data 1

Description of Additional Supplementary Files

Reporting Summary

## Data Availability

All relevant data supporting the findings of this study are available within the article and its Supplementary Information files or from the corresponding author upon request. The source data underlying Figs. 1c, d, 2b, d, 3c, d, g, i, 4c–e, 5b, d, f, 6b, d, and Supplementary Figs. 2a, b, d, e are provided as a Source Data file. Source data are provided with this paper.

## Source data

Source Data
