# Supplementary material for: Spt6 is a maintenance factor for centromeric CENP-A
Source: Nat Commun. 2020 Jun 10;11:2919. doi: 10.1038/s41467-020-16695-7 (PMC7287101; doi:10.1038/s41467-020-16695-7)
Supplement: Supplementary file 1 — Supplementary Information [file 41467_2020_16695_MOESM1_ESM.pdf]

Supplementary Figures

Title

Spt6 is a maintenance factor for centromeric CENP-A

Authors

Georg OM Bobkov[1,2], Anming Huang[3], Sebastiaan J.W. van den Berg[4,8], Sreyoshi Mitra[4,8], Eduard Anselm[1], Vasiliki Lazou[1], Sarah Schunter[5], Regina Feederle[6], Axel Imhof[7], Alexandra Lusser[3], Lars E.T. Jansen[8] and Patrick Heun[1]

6 supplementary figures with corresponding legends

Supplementary Fig. 1

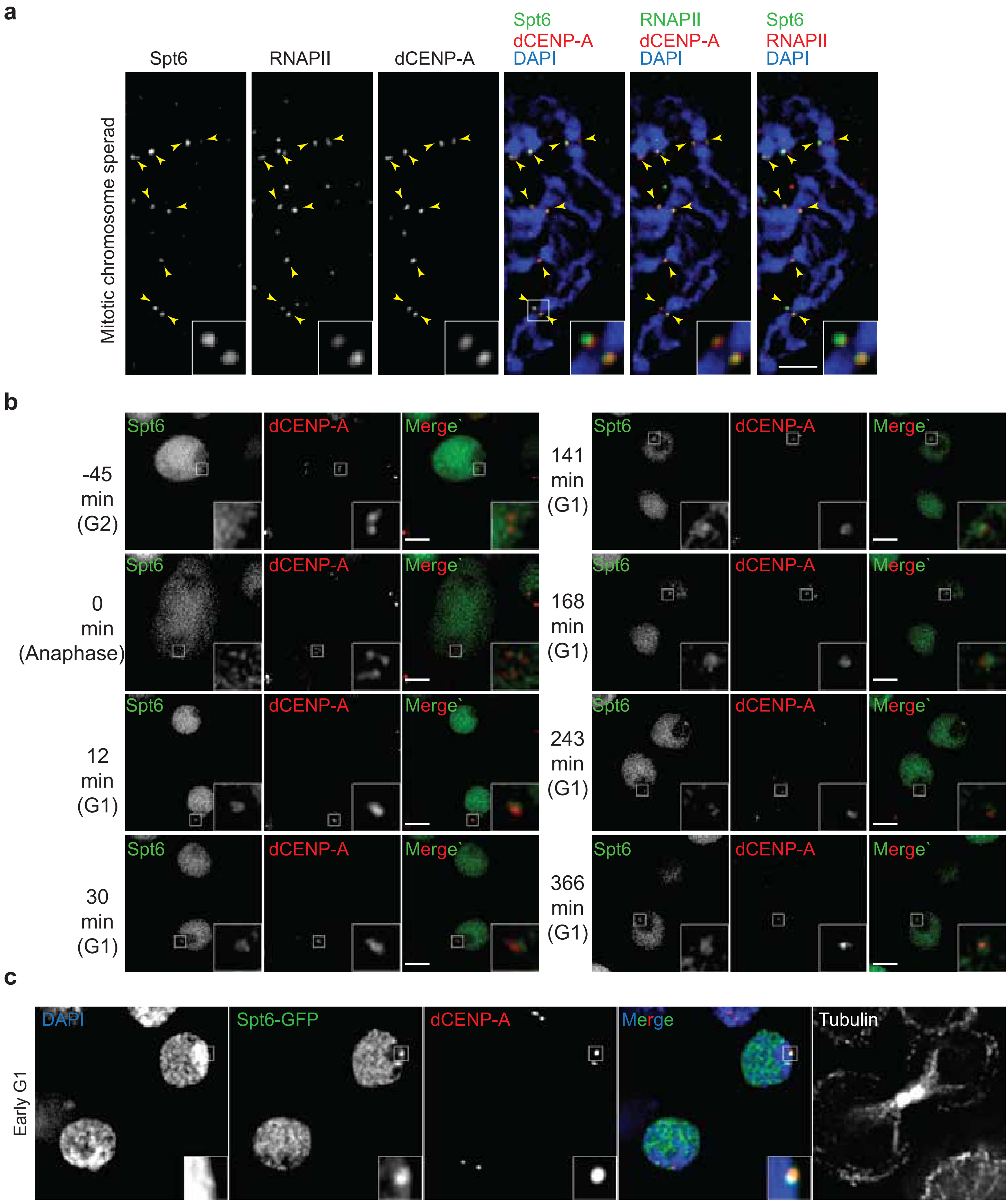

Supplementary Fig. 1 Spt6 localizes to centromeres from mitosis to G1. **a** A projection of fixed mitotic chromosome spreads immunostained for endogenous Spt6, RNAPII and dCENP-A is shown. Yellow arrow heads point to centromeres. N=3 independent experiments. **b** Single optical section of S2 cells are shown. Boxes indicate the 3x enlarged inset. Time-lapse live imaging pictures of cells transiently expressing Spt6-GFP and dCENP-A-mCherry. Numbers indicate minutes before/after onset of anaphase. Movies of 63 cells were taken in a cumulative way. **c** Single optical section of fixed early G1 cell expressing Spt6-GFP. dCENP-A immunodetection served as a marker of centromeres; tubulin staining allowed midbody detection. N=3 independent experiments. Scale bars represent 3 μm.

Supplementary Fig. 2

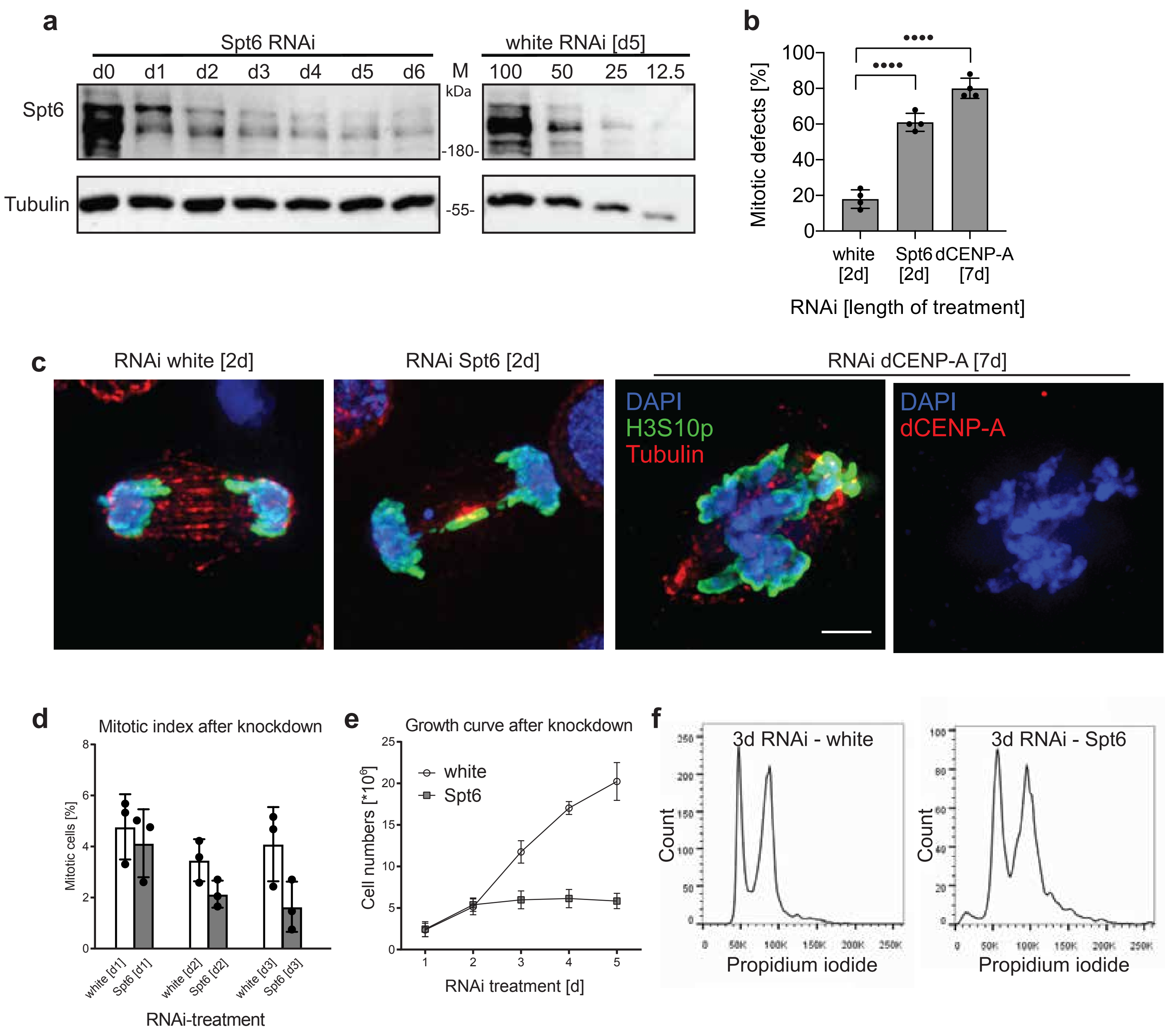

Supplementary Fig. 2 RNAi-mediated depletion of Spt6 results in mitotic defects and in an unspecific cell-cycle block. **a** Western Blot analysis of Spt6 protein levels in whole cell extracts after RNAi treatment. White RNAi served as a control. Spt6 detection by Western Blot resulted in two bands (five different antibodies tested), with the upper band likely representing a modified version of Spt6. M = marker. **b** Quantification of mitotic defects after white (control), Spt6 or dCENP-A RNAi. N=4 independent experiments; n=25 anaphase cells. Data are represented as mean + SD. Statistical significance: quadruple dots P<0.0001; n.s.=not significant (unpaired, two-tailed t-test). **c** Maximum intensity projection of representative mitotic cells for white, Spt6 and dCENP-A RNAi that were quantified in b. Scale bar represents 3 µm. **d** Mitotic index after control (white) or Spt6 RNAi. Data are represented as mean + SD. N=3 independent experiments, nwhite-RNAi = 230, nSpt6-RNAi= 122 cells. **e** 5d growth curve of S2 cells after RNAi-mediated depletion of control (white) or Spt6. N=3 independent experiments. Data are represented as mean +/-SD. **f** Single cell FACS profile of propidium iodide staining after 3d of white or Spt6 RNAi. FACS gating strategy is provided as Supplementary Fig. 7. Source data are provided as a Source Data file.

Supplementary Fig. 3

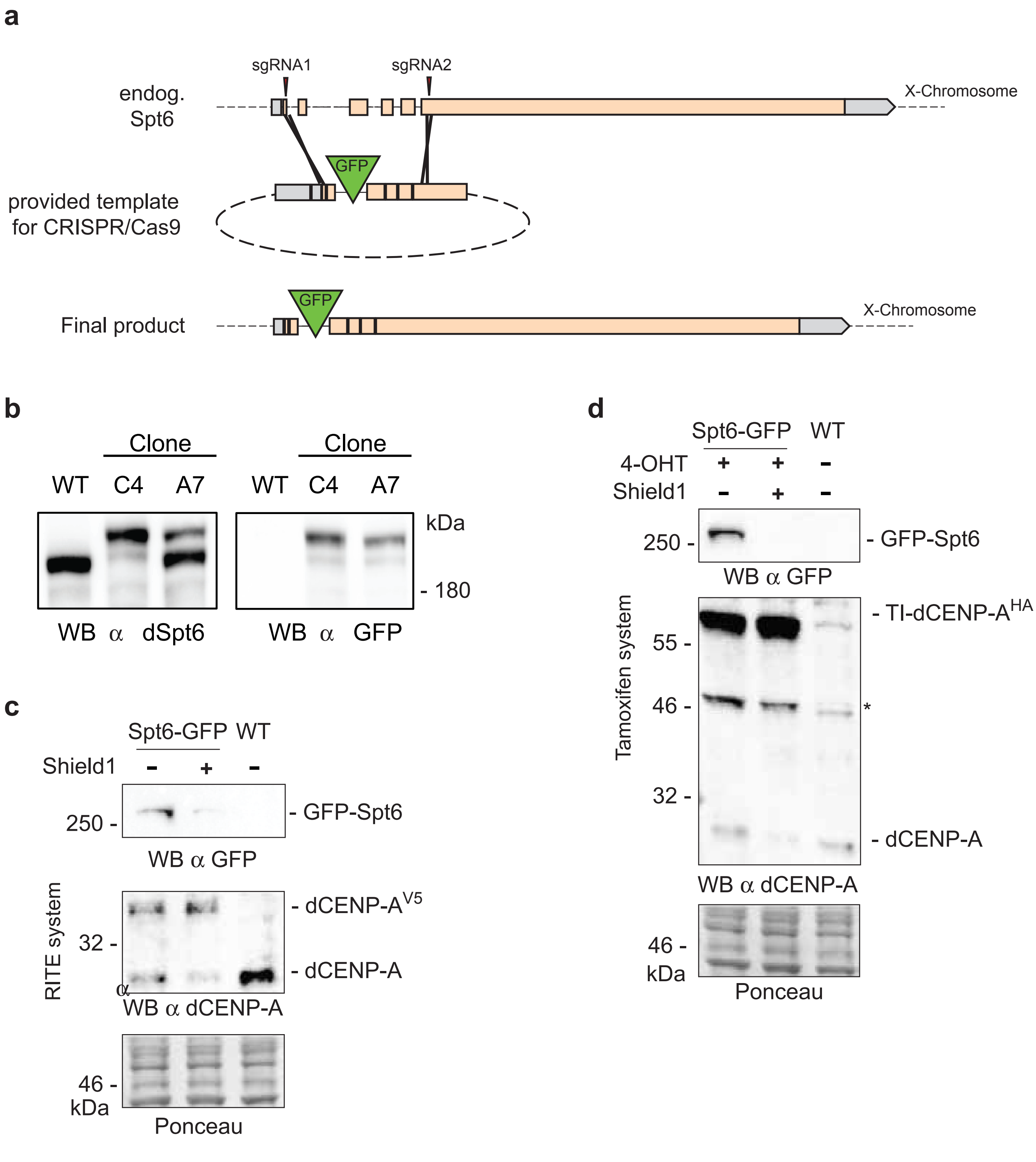

Supplementary Fig. 3 GFP-tagging of endogenous Drosophila Spt6 and dCENP-A levels in the RITE and Tamoxifen system. **a** Schematic representation of the GFP-Spt6 version created by CRISPR/Cas9. **b** Western blot analysis of Spt6 levels in whole cell extracts of wildtype (WT) cells and two clonal cell lines. Both alleles are tagged with GFP in clone C4, only one of the two genetic loci is tagged in clone A7. N=2 independent experiments. **c** Western blot analysis of endogenous and transgenic dCENP-A (dCENP-A^{V5}) levels in nuclear extracts of Spt6-GFP (clone C4) with and without Spt6 depletion (+/- Shield) and wildtype (WT) cells for the RITE system. N=3 independent experiments. Note, that endogenous dCENP-A adapts to lower levels in the presence of a constitutively expressed transgene (compare endogenous dCENP-A between Spt6-GFP and WT cell lines) similar to human cells as described in Foltz et al., 2006[3] (Fig. S1a, b). Western blot analysis for transgenic dCENP-A levels in the RITE system were performed for dCENP-A^{V5}, because Cre-recombinase treatment induces recombination to dCENP-A^{MYC} only in a subset of cells (about ≈20-40%). As the promoter remains unchanged, dCENP-A^{V5} levels can be used as a proxy for both transgenes. **d** Western blot analysis of endogenous and transgenic dCENP-A (TI-dCENP-A^{HA}) levels in nuclear extracts of Spt6-GFP (clone C4) with and without Spt6 depletion (+/- Shield) and wildtype (WT) cells for the Tamoxifen system in the presence of 4-OHT. N=3 independent experiments. Note the higher protein levels of TI-dCENP-A^{HA} in comparison to dCENP-A^{V5} and endogenous dCENP-A, allowing for a larger pool of new dCENP-A incorporation. Ponceau staining controls for similar loading. Source data are provided as a Source Data file.

Supplementary Fig. 4

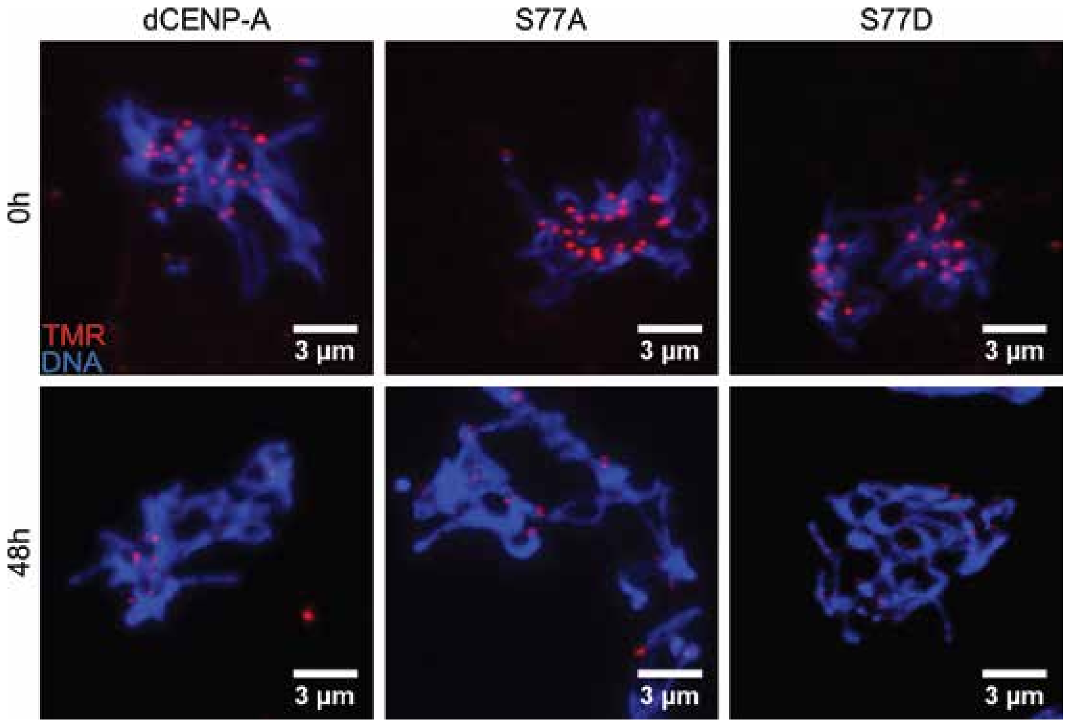

Supplementary Fig. 4 Representative images of mitotic chromosome spreads of S2 cells expressing low levels of SNAP-tagged wildtype, S77A and S77D dCENP-A right after TMR-staining (top panels) or after a chase period of 48h (bottom panels) are shown for one out of N=3 independent experiments. Note that images for S77D were subjected to 1.6x higher laser power to achieve signal intensities strong enough for quantification.

Supplementary Fig. 5

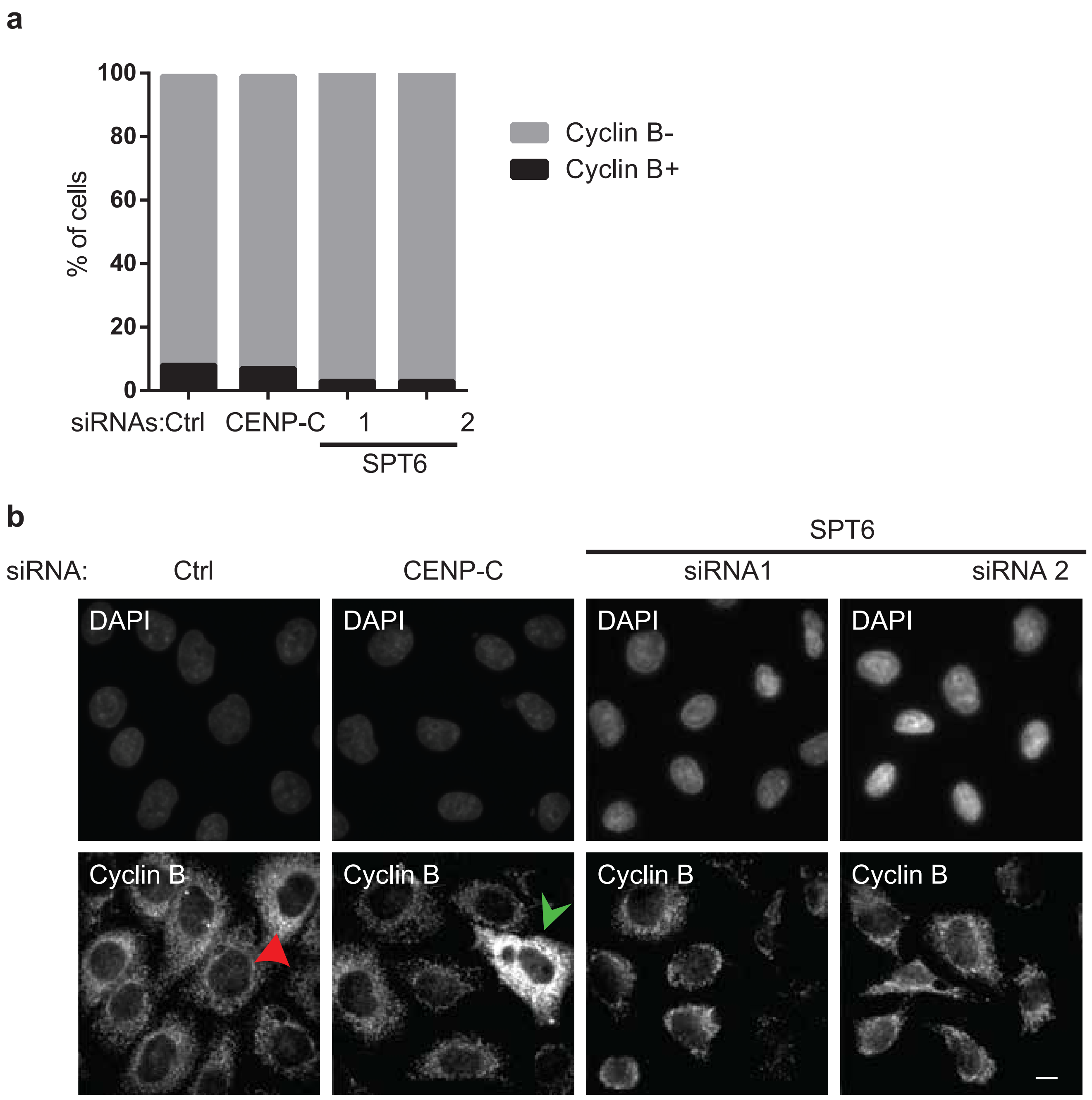

Supplementary Fig. 5 Cyclin B staining controls for cell synchronization and passage into G1. **a** Percentage of Cyclin B positive cells per siRNA condition. **b** Representative images of cells treated with indicated siRNAs stained for Cyclin B and DAPI. The green arrow head indicates a typical Cyclin B positive cell and the red arrow head a typical Cyclin B negative cell. N=3 independent experiments. Scale bar represents 10 μm.

Supplementary Fig. 6

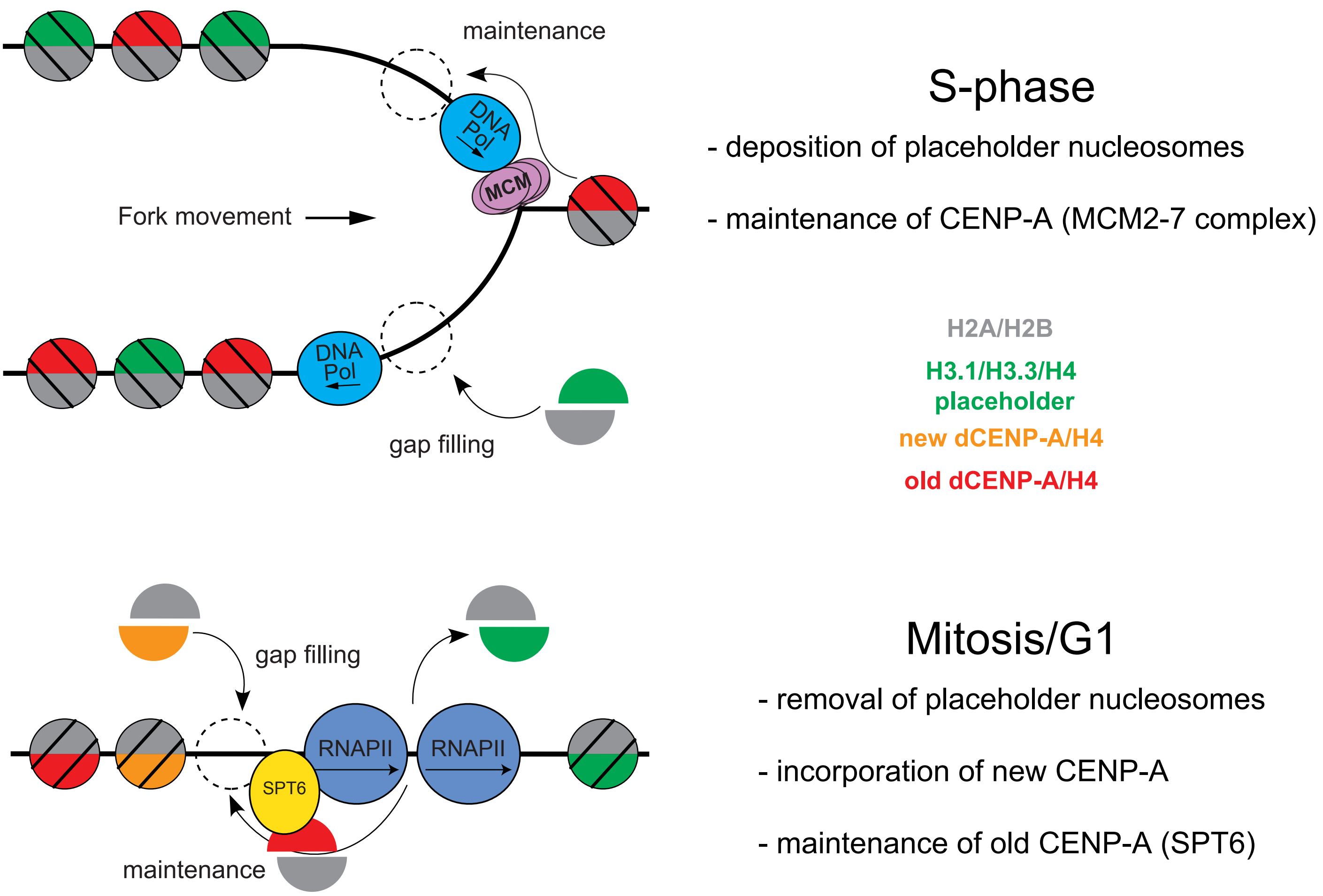

Supplementary Fig. 6 Schematic representation of nucleosome dynamics during the remodeling of centromeric chromatin in S-phase and Mitosis/G1.

Supplementary Figure 7

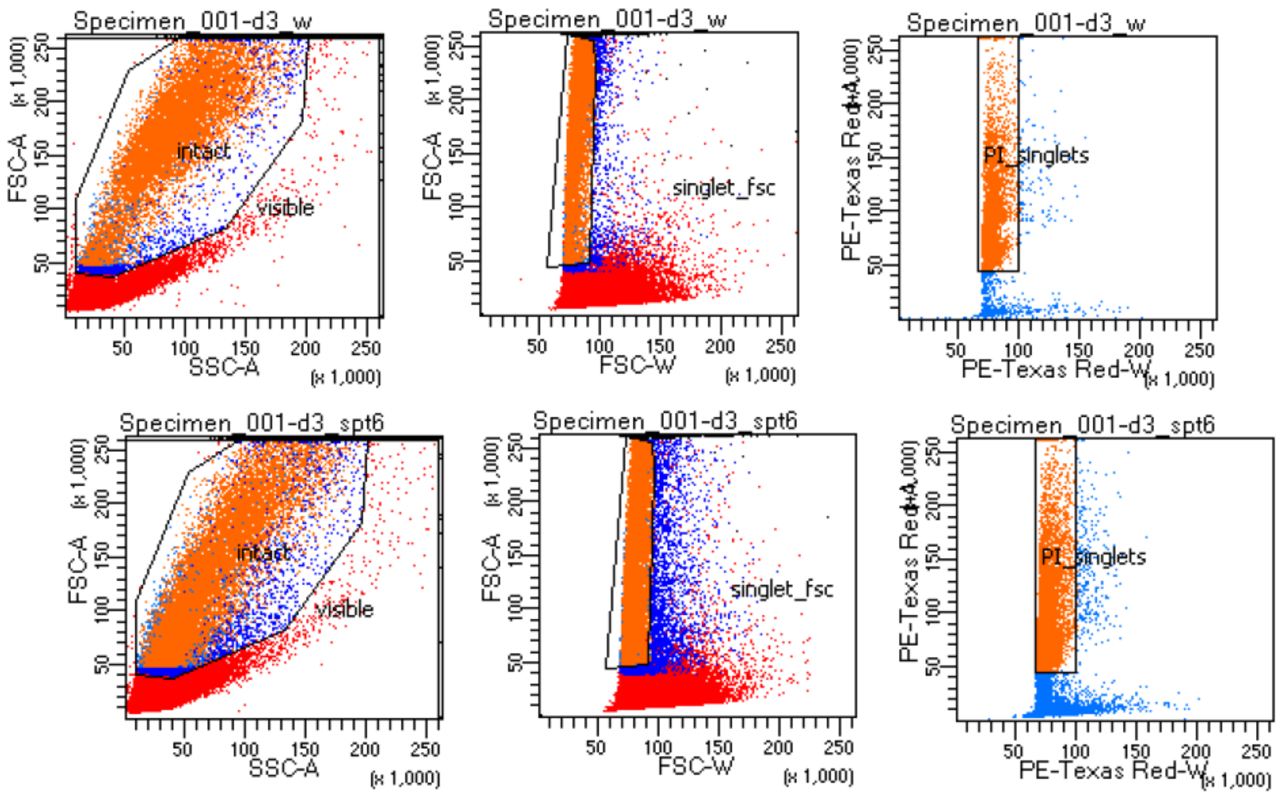

Supplementary Fig. 7 Gating strategy to generate FACS profile of propidium iodide stained S2 cells. FSC-A/SSC-A was used to separate intact cells from cell debris. Cell doublets were excluded from the analysis using both FSC-A/FSC-W and PE-Texas Red-A/PE-Texas Red-W measurements (refers to FACS graphs shown in Supplementary Fig. 2f).
