# Supplementary material for: Spt6 is a maintenance factor for centromeric CENP-A
Source: Nat Commun. 2020 Jun 10;11:2919. doi: 10.1038/s41467-020-16695-7 (PMC7287101; doi:10.1038/s41467-020-16695-7)
Supplement: Supplementary file 3 — Supplementary Data 1 [file 41467_2020_16695_MOESM3_ESM.pdf]

Supplemental information – Table 1: Primers, Plasmids and gBlocks

Primers and sgRNAs

	Cloning primers
dSpt6_F1	TCCGACTAGTATGGCCGAATTTCTGGACTCCGA
dSpt6_R2	GCATGATATCGTTCTCGTCGTAGAGGGGCGT
1kup_F_BstZ17I	ATAGTATACCAACAACAGGAGCACCGGACAC
2kup_R_NheI	CGAGCTAGCAATATAATGGAATAAAGCTTAACTTAATAATAAAGTGCG
dSpt6_R-AscI	ATATGGCGCGCCTAGATGTTCTCGTCGTAGAGGGGCGT
T2A_CD4_E1_F	CCGGAATTCGAGGGCAGAGGAAGTCTTCTAACATGCG
T2A_CD4_B1_R	AAAGGATCCCTAGCCGGTGTGGCGGCACCTGA
	RNAi primers
White fw (RNAi)	TTAATACGACTCACTATAGGGACTGCTCAATGGCCAACCTGTGGAC
White rev (RNAi)	TTAATACGACTCACTATAGGGCCTCGGCCATCAGAAGGATCTTGTC
Spt6_F(RNAi)	TTAATACGACTCACTATAGGGATGCCGGGCAAGTTCCTGCTGTCCTA
Spt6_R(RNAi)	TTAATACGACTCACTATAGGGGGCGTCATGAACGGAGTCTGTCCAC
CID fw (RNAi)	TTAATACGACTCACTATAGGGAGACCGTCGGCGAACAACTCAAAGT
CID rev (RNAi)	TTAATACGACTCACTATAGGGAGAAGCCGCTGCGTCAAGTACATCTC
Neg9	UACGACCGGUCUAUCGUAGTT
	sgRNA primers
dU6_2_sgRNA_F	GTTCGACTTGCAGCCTGAAATACGGCACG
dU6_2_3_sgRNA_R	AAAAAAGCACCGACTCGGTGCCACTTTTTCAAGTTGATAA
Spt6_sgRNA_5_fw	TTCGTGACGACTTCTCAAAGTACG
Spt6_sgRNA_5_rev	AACCGTACTTTGAGAAGTCGTCC
Spt6_sgRNA_9_fw	TTCGGTTACGATTCCGATGGCGT
Spt6_sgRNA_9_rev	AACACGCCATCGGAATCGTAACC
	Primers for Site-Directed Mutagenesis
S20A_F	CAACTCAAAGgCGCCGAACGACG
S20A_R	TTCGCCGACGGCCTGGGT
S75A_F	GAGAGACAGCgCCACCAGTGGCG
S75A_R	GCTGCCGGCTGCCTCCGT
S77A_F	CAGCTCCACCgcTGGCGAGGAGGAGGACC
S77A_R	TCTCTCGCTGCCGGCTGC
B75AM77A_F	CAGCgCCACCgcTGGCGAGGAGGAGGAC
S20D_F	CAACTCAAAGgacCCGAACGACGACGACACGGCC

S20D_R	TTCGCCGACGGCCTGGGT
S75A_F	GAGAGACAGCgaCACCAGTGGCGAGGAGGAGG
S75A_R	GCTGCCGGCTGCCTCCGT
S77D_F	CAGCTCCACCgacGGCGAGGAGGAGGACC
S77D_R	TCTCTCGCTGCCGGCTGC
B75DM77D_F	CGAGAGACAGCgacACCgacGGCGAGGAGGAGGACC
B75DM77D_R	GGTCCTCCTCCTCGCCgtcGGTGTCGCTGTCTCTCG

Plasmids:

pMT-CID-GFP-hygro	Heun et al., 2006[1]
pMT-Spt6-GFP-hygro	This study
pIB-resSpt6-stop	This study
pMT_FKBP_L106P_Fbox_GFPbinder_hygro_opt	This study
pMT-CID-HA-ERT2_kana_opt	This study
pRITE_MT_CID_V5_puro_3myc_CD4	This study
1kup_resGFPSpt6_stop	This study
pIB_Cas9_CD4_Blast	
pET-His6-Sumo_Spt6199-338	This study
pET22b_H3	gift from K. Luger
pET22b_H4	gift from K. Luger
pMT-SNAP-CENP-A	Boltengagen et al., 2016[2]
pMT-SNAP-CENP-A_S77D	This study
pMT-SNAP-CENP-A_S20/75/77D	This study

gBlocks

resGFPSpt6_HindIII_Eco47III
GTTAAGCTTTATTCCATTATATTGCTAGTTATGGCCGAATTTCTGGACTCCGA
GGCGGAAGAATCGGAGGAGGAGGAAGAATTGGACGTCAACGAGCGCAAGA
GACTCAAGAAACTGAAAGCAGCCGTCTCCGATAGTAGCGAAGAAGAAGAAG
GTGAGTTTTTCCACTGGAGCCGCTGAAGGCGCGTACAAATACATACATACAT
ACATACGTATGTGGCGATCTCTGGCGGGTTTTTCACCCCTCTCACCAGGCG
CGCCAGATCCACACATGGTGAGCAAGGGCGAGGAGCTGTTCACCGGGGTG
GTGCCCATCCTGGTCGAGCTGGACGGCGACGTAAACGGCCACAAGTTCAG
CGTGTCCGGCGAGGGCGAGGGCGATGCCACCTACGGCAAGCTGACCCTG

AAGTTCATCTGCACCACCGGCAAGCTGCCCGTGCCCTGGCCCACCCTCGT
GACCACCCTGACCTACGGCGTGCAGTGCTTCAGCCGCTACCCCGACCACA
TGAAGCAGCACGACTTCTTCAAGTCCGCCATGCCCGAAGGCTACGTCCAGG
AGCGCACCATCTTCTTCAAGGACGACGGCAACTACAAGACCCGCGCCGAG
GTGAAGTTCGAGGGCGACACCCTGGTGAACCGCATCGAGCTGAAGGGCAT
CGACTTCAAGGAGGACGGCAACATCCTGGGGCACAAGCTGGAGTACAACT
ACAACAGCCACAACGTCTATATCATGGCCGACAAGCAGAAGAACGGCATCA
AGGTGAACTTCAAGATCCGCCACAACATCGAGGACGGCAGCGTGCAGCTC
GCCGACCACTACCAGCAGAACACCCCCATCGGCGACGGCCCCGTGCTGCT
GCCCGACAACCACTACCTGAGCACCCAGTCCGCCCTGAGCAAAGACCCCA
ACGAGAAGCGCGATCACATGGTCCTGCTGGAGTTCGTGACCGCCGCCTCG
ACTCGAGGTACCTGGTAACAGTTTTCAGTTGCATATGTATATCTGCGGCTAC
AATGAAGGTAAAAGGTAAACCGATATTCTTATTGCCCTATTCCGTAGACGAC
GAGGAACGGCTGCGCGAAGAGCTCAAAGATCTGATCGACGACAATCCCATC
GAGGAGGACGATGGCAGTGGTTACGATTCCGACGGAGTGGGCAGCGGCAA
GAAGCGCAAAAAACATGAAGACGACGATCTGGACGATCGACTGGAGGACG
ACGACTACGATCTTATCGAGGAGAATCTGGGCGTCAAAGTCGAGAGACGGA
AGCGGTTCAAGCGTCTGCGTCGCATCCACGACAACGAAAGCGATGGCGAG
GAGCAGCACGTGGACGAGGGTCTGGTGCGTGAGCAGATTGCTGAACAATT
GTTCGACGAGAACGATGAGAGCATTGGTCATCGCAGCGAGCGCAGTCACA
GGGAGGCGGACGACTACGACGACGTGGATACGGAGTCGGACGCAGATGA
CTTCATTGTGGACGATAATGGGCGTCCCATAGCCGAGAAGAAGAAGAAACG
GCGCCCCATCTTTACAGACGCATCACTGCAGGAAGGTCAGGACATCTTTGG
CGTAGACTTCGACTATGACGACTTCTCAAAATATGAAGAAGACGACTACGAA
GATGACTCGGAAGGAGATGAGTACGATGAGGATCTGGGCGTCGGCGATGA
TACGCGCGTCAAGAAGAAGAAAGCGCTCAA

GFP, Mutated sgRNA9 and sgRNA5 target regions (respectively)

dSpt6_199-338_Ecoli_codopt_biobrick_IDT
TACTTCCAATCCAATGCAATGACCGATGCGTCTCTGCAAGAAGGTCAAGATA
TTTTTGGCGTGGATTTTGATTATGATGATTTTAGCAAATATGAAGAAGACGAT
TATGAAGATGACAGCGAAGGCGATGAATATGATGAAGATCTGGGCGTGGGC
GACGATACCCGCGTGAAAAAGAAGAAAGCGCTGAAAAAGAAAGTGGTGAAG
AAAACCATTTTTGATATTTATGAACCGAGCGAACTGAAACGCGGCCATTTTA
CCGATATGGATAACGAAATTCGCAAAACCGATATTCCGGAACGCATGCAGC
TGCGCGAAGTGCCGGTGACCCCGGTGCCGGAAGGCAGCGATGAACTGGAT
CTGGAAGCGGAATGGATTTATAAATATGCGTTTTGCAAACATACCGTGAGCG
AACAGGAAAAACCGGAAAGCCGCGAAAAATAATAACATTGGAAGTGGATAA

Biobrick

FKBP_L106P degron
AAAGGTACCAACATGGGTGTGCAGGTTGAGACGATCTCGCCCGGTGATGGT
CGCACCTTCCCCAAGCGTGGACAGACCTGCGTGGTCCATTACACTGGCATG
CTGGAGGATGGTAAGAAGGTGGACTCCTCGCGTGACCGTAACAAGCCATTC

AAGTTCATGCTGGGCAAGCAGGAGGTGATTCGCGGTTGGGAGGAGGGTGT
CGCCCAGATGTCCGTGGGCCAACGCGCCAAGCTGACCATCAGCCCAGACT
ATGCTTACGGCGCCACTGGACACCCCGGTATCATCCCACCCCACGCCACCT
TGGTGTTCGACGTGGAGCTGCTGAAGCCGGAGGGCGGAGGCAGCGGAGG
TGGAAGCTCCGGCGGACTAGTAAC

FKBP_L106P

F-box-GFP binder
TCCACTAGTAACGGCCGCCAGTGTGCTGGAATTCAAAATGATGAAAATGGA
GACTGACAAAATAATGGACGAAACCAACTCCAATGCACAGGCCTTCACAAC
CACTATGCTGTACGACCCGGTGCGCAAGAAAGACTCATCGCCCACCTACCA
AACGGAGCGGGAACTCTGCTTTCAGTACTTCACCCAGTGGAGCGAGTCGG
GCCAGGTGGACTTTGTGGAGCACCTGCTGTCGCGCATGTGCCACTATCAAC
ACGGACAGATCAATGCCTATCTCAAGCCGATGCTCCAGCGGGACTTTATCA
CATTGCTGCCAATCAAGGGTCTGGATCACATCGCAGAAAACATTTTGTCGTA
CTTGGATGCCGAATCGCTCAAATCATCCGAGCTGGTCTGCAAGGAATGGCT
GCGCGTCATTTCCGAGGGCATGCTCTGGAAGAAGCTCATCGAACGCAAGGT
GCGCACAGATTCCTTGTGGCGCGGACTGGCCGAGCGGCGTAATTGGATGC
AGTACCTCTTCAAGCCAAGACCGGGCCAGACTCAACGGCCACACTCATTCC
ATCGCGAGTTGTTCCCCAAGATAATGAATGACATTGACAGCATAGAGAACAA
CTGGCGGACTGGCCGCCACCTCGAGATGGATCAAGTCCAACTGGTGGAGT
CTGGTGGCGCTTTGGTGCAGCCAGGTGGCTCTCTGCGTTTGTCCTGTGCCG
CTTCTGGCTTCCCAGTGAACCGCTATTCCATGCGCTGGTATCGCCAGGCTC
CAGGCAAAGAGCGTGAGTGGGTAGCCGGTATGTCCAGCGCGGGTGATCGT
AGCTCCTATGAAGACTCCGTGAAGGGCCGTTTCACCATCAGCCGTGACGAT
GCCCGTAACACGGTGTATCTGCAAATGAACAGCTTGAAACCTGAAGATACG
GCCGTGTATTACTGTAATGTGAACGTGGGCTTCGAGTATTGGGGCCAAGGC
ACCCAGGTCACCGTCTCCAGCTAATCTAGAGGG

NSlmb (Fbox protein), GFP binder (GBP)

DNA sequences assembled from multiple gBlocks and PCR products

pRITE
AGTGGCGGCCGCTCGAGCGGTGGCTCCGGCGGAAGCATAACTTCGTATAA
TGTATGCTATACGAAGTTATCACCGCGGTTCGAAGGTAAGCCTATCCCTAAC
CCTCTCCTCGGTCTCGATTCTACCCGTACCGGTGAGGGCAGAGGAAGTCTT
CTAACATGCGGTGACGTGGAGGAGAATCCCGGCCCTGCCAGCGGTAGCGG
CAGCGGCAACATGACCGAGTACAAGCCCACCGTGCGTCTGGCCACCCGTG
ACGATGTGCCACGTGCCGTTCGCACCCTGGCCGCCGCCTTCGCCGACTAT
CCAGCCACCCGTCACACCGTGGACCCAGATCGCCACATCGAGCGTGTCAC
TGAGCTGCAGGAGCTGTTCCTGACCCGCGTTGGTCTGGACATCGGCAAGG
TGTGGGTGGCTGACGATGGCGCTGCCGTGGCTGTGTGGACTACCCCAGAA
TCCGTGGAGGCCGGAGCCGTGTTCGCCGAGATCGGCCCACGTATGGCCGA
GCTGTCGGGCTCGCGTCTCGCTGCCCAGCAGCAGATGGAGGGACTCCTGG

CCCCACACCGTCCCAAGGAGCCCGCCTGGTTCCTCGCCACCGTGGGTGTG
TCCCCAGATCACCAGGGCAAGGGCCTGGGCTCCGCCGTCGTTCTGCCGGG
CGTCGAGGCCGCCGAGCGCGCTGGAGTCCCCGCCTTCCTGGAGACCTCG
GCCCCCCGTAACCTGCCCTTCTATGAGCGTCTGGGATTCACCGTCACCGCC
GACGTTGAGGTTCCCGAGGGACCCCGCACCTGGTGCATGACCCGCAAGCC
CGGTGCCTGAACGCGTCCGCTGATCAGCCTCGACTGTGCCTTCTAAGATCC
AGACATGATAAGATACATTGATGAGTTTGGACAAACCACAACTAGAATGCAG
TGAAAAAAATGCTTTATTTGTGAAATTTGTGATGCTATTGCTTTATTTGTAACC
ATTATAAGCTGCAATAAACAAGTTAACAACAACAATTGCATTCATTTTATGTTT
CAGGTTCAGGGGGAGGTGTGGGAGGTTTTTTAAAGCAAGTAAAACCTCTAC
AAATGTGGTATGGCTGATTATGATCAATAACTTCGTATAATGTATGCTATACG
AAGTTATCAAGATCTGCTAGCATGGCACCCGGGGAGCAGAAACTCATTAGT
GAGGAGGACTTGGGCAGTGGCAGTGGAGGAGAACAAAAGCTCATCTCGGA
GGAGGACCTGGGTTCCGGCTCCGGCGGAGAACAGAAGTTGATCTCGGAGG
AGGACCTGGAATTCGAGGGCAGAGGAAGTCTTCTAACATGCGGTGACGTG
GAGGAGAATCCCGGCCCTGCTAGTGGTAGCGGCAGCGGTAGCATGAACCG
GGGAGTCCCTTTTAGGCACTTGCTTCTGGTGCTGCAACTGGCGCTCCTCCC
AGCAGCCACTCAGGGAAAGAAAGTGGTGCTGGGCAAAAAAGGGGATACAG
TGGAACTGACCTGTACAGCTTCCCAGAAGAAGTCCATACAATTCCACTGGAA
AAACTCCAACCAGATAAAGATTCTGGGAAATCAGGGCTCCTTCTTAACTAAA
GGTCCATCCAAGCTGAATGATCGCGCTGACTCAAGAAGAAGCCTTTGGGAC
CAAGGAAACTTCCCCCTGATCATCAAGAATCTTAAGATAGAAGACTCAGATA
CTTACATCTGTGAAGTGGAGGACCAGAAGGAGGAGGTGCAATTGCTAGTGT
TCGGATTGACTGCCAACTCTGACACCCACCTGCTTCAGGGGCAGAGCCTGA
CCCTGACCTTGGAGAGCCCCCCTGGTAGTAGCCCCTCAGTGCAATGTAGGA
GTCCAAGGGGTAAAAACATACAGGGGGGGAAGACCCTCTCCGTGTCTCAG
CTGGAGCTCCAGGATAGTGGCACCTGGACATGCACTGTCTTGCAGAACCAG
AAGAAGGTGGAGTTCAAAATAGACATCGTGGTGCTAGCTTTCCAGAAGGCC
TCCAGCATAGTCTATAAGAAAGAGGGGGAACAGGTGGAGTTCTCCTTCCCA
CTCGCCTTTACAGTTGAAAAGCTGACGGGCAGTGGCGAGCTGTGGTGGCA
GGCGGAGAGGGCTTCCTCCTCCAAGTCTTGGATCACCTTTGACCTGAAGAA
CAAGGAAGTGTCTGTAAAACGGGTTACCCAGGACCCTAAGCTCCAGATGGG
CAAGAAGCTCCCGCTCCACCTCACCCTGCCCCAGGCCTTGCCTCAGTATGC
TGGCTCTGGAAACCTCACCCTGGCCCTTGAAGCGAAAACAGGAAAGTTGCA
TCAGGAAGTGAACCTGGTGGTGATGAGAGCCACTCAGCTCCAGAAAAATTT
GACCTGTGAGGTGTGGGGACCCACCTCCCCTAAGCTGATGCTGAGCTTGAA
ACTGGAGAACAAGGAGGCAAAGGTCTCGAAGCGGGAGAAGGCGGTGTGG
GTGCTGAACCCTGAGGCGGGGATGTGGCAGTGTCTGCTGAGTGACTCGGG
ACAGGTCCTGCTGGAATCCAACATCAAGGTTCTGCCCACATGGTCGACCCC
GGTGCAGCCAATGGCCCTGATTGTGCTGGGGGGCGTCGCCGGCCTCCTGC
TTTTCATTGGGCTAGGCATCTTCTTCTGTGTCAGGTGCCGCCACACCGGCTA
GGGATCCGTTGTTTAAACCCG

loxP sites, V5, T2A, puromycin resistance, 3xMYC, CD4

1. Heun P, Erhardt S, Blower MD, Weiss S, Skora AD, Karpen GH. Mislocalization of the Drosophila Centromere-Specific Histone CID Promotes Formation of Functional Ectopic Kinetochores. *Dev Cell* **10**, 303-315 (2006).

2. Boltengagen M, *et al.* A novel role for the histone acetyltransferase Hat1 in the CENP-A/CID assembly pathway in Drosophila melanogaster. *Nucleic Acids Res* **44**, 2145-2159 (2016).

3. Foltz DR, Jansen LE, Black BE, Bailey AO, Yates JR, 3rd, Cleveland DW. The human CENP-A centromeric nucleosome-associated complex. *Nat Cell Biol* **8**, 458-469 (2006).
