# Supplementary material for: Spt6 is a maintenance factor for centromeric CENP-A
Source: Nat Commun. 2020 Jun 10;11:2919. doi: 10.1038/s41467-020-16695-7 (PMC7287101; doi:10.1038/s41467-020-16695-7)
Supplement: Supplementary file 4 — Description of Additional Supplementary Files [file 41467_2020_16695_MOESM4_ESM.pdf]

Description of Additional Supplementary Files

File: Supplementary Table 1:

List of primers, siRNAs, sgRNAs, and gBlock fragments including DNA sequences and list of all plasmids used.

File: Source Data

Contains the tabulated data extracted from image quantification for histogram plots in Figures 1-3, 5,6, Supplemental Figures 2, 3 and the raw images for all immunoblots presented in Figures 2, 4, 6 and Supplemental Figures 2, 3.

File: FACS Gating Strategy

Image exemplifies the FACS gating strategy.
