# Supplementary material for: Spt6 is a maintenance factor for centromeric CENP-A
Source: Nat Commun. 2020 Jun 10;11:2919. doi: 10.1038/s41467-020-16695-7 (PMC7287101; doi:10.1038/s41467-020-16695-7)
Supplement: Supplementary file 5 — Reporting Summary [file 41467_2020_16695_MOESM5_ESM.pdf]

nature research

Corresponding author(s): Patrick Heun

Last updated by author(s): Mar 9, 2020

Reporting Summary

Nature Research wishes to improve the reproducibility of the work that we publish. This form provides structure for consistency and transparency in reporting. For further information on Nature Research policies, see Authors & Referees and the Editorial Policy Checklist.

Statistics

For all statistical analyses, confirm that the following items are present in the figure legend, table legend, main text, or Methods section.

n/a	Confirmed	
☐	☒	The exact sample size (*n*) for each experimental group/condition, given as a discrete number and unit of measurement
☐	☒	A statement on whether measurements were taken from distinct samples or whether the same sample was measured repeatedly
☐	☒	The statistical test(s) used AND whether they are one- or two-sided<br>*Only common tests should be described solely by name; describe more complex techniques in the Methods section.*
☒	☐	A description of all covariates tested
☒	☐	A description of any assumptions or corrections, such as tests of normality and adjustment for multiple comparisons
☐	☒	A full description of the statistical parameters including central tendency (e.g. means) or other basic estimates (e.g. regression coefficient) AND variation (e.g. standard deviation) or associated estimates of uncertainty (e.g. confidence intervals)
☐	☒	For null hypothesis testing, the test statistic (e.g. *F*, *t*, *r*) with confidence intervals, effect sizes, degrees of freedom and *P* value noted<br>*Give P values as exact values whenever suitable.*
☒	☐	For Bayesian analysis, information on the choice of priors and Markov chain Monte Carlo settings
☒	☐	For hierarchical and complex designs, identification of the appropriate level for tests and full reporting of outcomes
☒	☐	Estimates of effect sizes (e.g. Cohen's *d*, Pearson's *r*), indicating how they were calculated

Our web collection on statistics for biologists contains articles on many of the points above.

Software and code

Policy information about availability of computer code

Data collection	softWoRx Explorer Suite (Applied Precision) 2.0, Image Lab 6.1 (BIO RAD), BD FACScalibur Flow Cytometer
Data analysis	softWoRx Explorer Suite (Applied Precision) 2.0, CRaQ (Bodor et al., 2012), GraphPad Prism 8.4.0, Imaris V5.1, FlowJo

For manuscripts utilizing custom algorithms or software that are central to the research but not yet described in published literature, software must be made available to editors/reviewers. We strongly encourage code deposition in a community repository (e.g. GitHub). See the Nature Research guidelines for submitting code & software for further information.

Data

Policy information about availability of data

All manuscripts must include a data availability statement. This statement should provide the following information, where applicable:

- Accession codes, unique identifiers, or web links for publicly available datasets
- A list of figures that have associated raw data
- A description of any restrictions on data availability

All relevant data supporting the findings of this study are available within the article and its Supplementary Information files or from the corresponding author upon request. The source data for Figs. 1, 2, 3, 4 and 6 and Supplementary Figs. 2 and 4 are provided as a Data source files 1 and 2, which includes all image quantification data and raw immunoblots, respectively.

October 2018

Field-specific reporting

Please select the one below that is the best fit for your research. If you are not sure, read the appropriate sections before making your selection.

☒ Life sciences ☐ Behavioural & social sciences ☐ Ecological, evolutionary & environmental sciences

For a reference copy of the document with all sections, see nature.com/documents/nr-reporting-summary-flat.pdf

Life sciences study design

All studies must disclose on these points even when the disclosure is negative.

Sample size	Sample sizes of 15 to 69 were taken for each experiment for Fig. 1c, 2d, 3c,d,g,i. This number allowed to test the null-hypothesis in the paper and achieved p values between control and experiment that were either clearly significantly different or not. Samples for live imaging in 1d was 23 and 39 were taken in a cumulative way. For Fig. 4d and Suppl. Fig. 4a sample sizes of >1000 were taken per experiment. For Fig. 6b, d Sample sizes of 110-196 were taken in cell lines stably expressing dCENP-A mutants. This numbers again allowed to assign a clear statistical difference between control and experiment. For Fig. 6f, sample size was 250-368 from 10-15 cells. RNAi experiments in Suppl. Fig. 2b and 2d was 25 for each experiment and condition. Suppl. Fig. 2d had a sample size of 122-230 mitotic cells. Sample size for Suppl. Fig. 2e was 2.0 -23.5 x10^6 cells.
Data exclusions	Images where the integrity of the nucleus has been compromised based on DAPI stain have been omitted from the analysis based. This includes small over-condensed DAPI "bodies" as well as fragmented nuclei.
Replication	All experiments shown have been successfully replicated at least 3 times, except for the time-lapse experiments shown in Fig. 1c. Since time-lapse experiments stretch over several days we decided to not separate them into individual experiments and compare to each other, but accumulate a single larger data set.
Randomization	Samples were allocated into experimental groups based on the same experimental setting. We did not perform any randomization. Potential covariants were controlled for by checking for maintained nuclear integrity (Fig. 1,2,3,4,6), or on effects on related proteins (Fig. 4c and Suppl Fig. 4a CENP-B).
Blinding	Blinding was not undertaken as most of the experiments contain a second marker that in the image channels that clearly identify the cohort. For example, measuring new and old CENP-A levels with and without removal of GFP-Spt6 clearly identifies each experimental setting due to the absence or presence of GFP-Spt6 respectively in the green channel.

Reporting for specific materials, systems and methods

We require information from authors about some types of materials, experimental systems and methods used in many studies. Here, indicate whether each material, system or method listed is relevant to your study. If you are not sure if a list item applies to your research, read the appropriate section before selecting a response.

Materials & experimental systems

n/a	Involved in the study	
☐	☒	Antibodies
☐	☒	Eukaryotic cell lines
☒	☐	Palaeontology
☒	☐	Animals and other organisms
☒	☐	Human research participants
☒	☐	Clinical data

Methods

n/a	Involved in the study	
☒	☐	ChIP-seq
☐	☒	Flow cytometry
☒	☐	MRI-based neuroimaging

Antibodies

Antibodies used	Unless otherwise noted, all antibodies were used 1:100 diluted: chicken α dCENP-A (1:20; P. Heun), rat α dCENP-A (4F8; E. Kremmer), rabbit α H3S10p (Abcam), mouse α dSpt6 (26D12, IgG2b, 1:50), rat α dSpt6 (13D4, IgG1; 1:50), mouse α GFP (Clone 496; D. van Essen), mouse α tubulin (Sigma) and mouse α V5 (Invitrogen). Secondary antibodies coupled to Alexa 488, Alexa 555 and Alexa 647 fluorophores (Invitrogen) were used at a 1:100 dilution. Counterstaining of DNA was performed by DAPI (5 µg/ml; 3’). Antibodies against CENP-B (Santa Cruz Biotechnology, sc-22788 Rabbit polyclonal) and cyclin B1 (sc-245; Santa Cruz Biotechnology) were used at dilutions of 1:1000 and 1:50. Fluorescent secondary antibodies Donkey anti-Rabbit FITC and Donkey anti-mouse 680 (Rockland) were used at a dilution of 1:200.
Validation	All primary antibodies have been validate for specificity by immunofluorescence combined with a counterstain of the relevant compartment (e.g. centromeres) or Western blots testing for proteins of the right size (or both). In the case of the Spt6 antibody, disappearance of the relevant double band in the Western blot has been verified when GFP-Spt6 (as the only copy) was removed.

Eukaryotic cell lines

Policy information about cell lines

Cell line source(s)	All Drosophila tissue culture work is based on the Schneider S2 cell clone L2-4. The original source was the Botchan lab, Berkeley. Human cell lines were HeLa cells.
Authentication	To our knowledge none of the cell lines used were authenticated.
Mycoplasma contamination	The original wildtype S2 cell line from which subclones have been produced for this paper has been tested and are mycoplasm free. The Hela cells are mycoplasm free.
Commonly misidentified lines (See ICLAC register)	*Name any commonly misidentified cell lines used in the study and provide a rationale for their use.*

Flow Cytometry

Plots

Confirm that:

☒ The axis labels state the marker and fluorochrome used (e.g. CD4-FITC).

☒ The axis scales are clearly visible. Include numbers along axes only for bottom left plot of group (a 'group' is an analysis of identical markers).

☒ All plots are contour plots with outliers or pseudocolor plots.

☒ A numerical value for number of cells or percentage (with statistics) is provided.

Methodology

Sample preparation	Cells were pelleted in a FACS tube (7', 1000xg) and fixed at 4°C overnight in 70% ethanol. Fixed cells were stained for 1h in the dark (50 µg/ml propidium iodide, 100 µg/ml RNase in PBS) and directly subjected to analysis on a BD FACScalibur Flow Cytometer using a gate for single cells.
Instrument	BD FACScalibur Flow Cytometer
Software	The FACS data was analysed with FlowJo
Cell population abundance	Sorting was not performed
Gating strategy	FSC/SSC was used to separate single cells from cell debris and doublets.

☐ Tick this box to confirm that a figure exemplifying the gating strategy is provided in the Supplementary Information.
