# Supplementary material for: Spt6 is a maintenance factor for centromeric CENP-A
Source: Nat Commun. 2020 Jun 10;11:2919. doi: 10.1038/s41467-020-16695-7 (PMC7287101; doi:10.1038/s41467-020-16695-7)
Supplement: Supplementary file 2 — Peer Review File [file 41467_2020_16695_MOESM2_ESM.docx]

Peer Review Information

**Journal:** Nature Communications

**Manuscript title:** Spt6 is a maintenance factor for centromeric CENP-A

**Corresponding author name(s):** Dr Patrick Heun

**Editorial notes:**

**Transferred manuscripts**

This manuscript has been previously reviewed at another journal that is not operating a transparent peer review scheme. This document only contains reviewer comments and rebuttal letters for versions considered at *Nature Communications*.

**Transferred manuscripts (no peer review at *Nature Communications*)**

This manuscript has been previously reviewed at another journal that is not operating a transparent peer review scheme. The manuscript was considered suitable for publication without further review at *Nature Communications*.

**Redactions – transferred manuscripts (mention of previous referee reports from elsewhere)**

This manuscript has been previously reviewed at another journal that is not operating a transparent peer review scheme. This document only contains reviewer comments and rebuttal letters for versions considered at *Nature Communications*. Mentions of prior referee reports have been redacted.

**Redactions – transferred manuscripts (mention of the other journal)**

This manuscript has been previously reviewed at another journal that is not operating a transparent peer review scheme. This document only contains reviewer comments and rebuttal letters for versions considered at *Nature Communications*. Mentions of the other journal have been redacted.

**Redactions – unpublished data**

Parts of this Peer Review File have been redacted as indicated to maintain the confidentiality of unpublished data.

**Redactions – confidential patient information**

Parts of this Peer Review File have been redacted as indicated to maintain patient confidentiality.

**Redactions – published data**

Parts of this Peer Review File have been redacted as indicated to remove third-party material where no permission to publish could be obtained.

**Amendments – published data**

[Figure X/The figure on page X] in this Peer Review File has been amended to remove third-party material where no permission to publish could be obtained.

**Published data (credit line for image)**

[Figure X/The figure on page X] in this Peer Review File is reproduced with permission from [Copyright Holder].

**Redactions – reviewer opt-out**

Parts of this Peer Review File have been redacted as indicated as we could not obtain permission to publish the reports of reviewer ###.

**Reviewer comments in marked-up manuscript**

In their review of the [first/second/third/...] version of this manuscript, reviewer ### added their comments to the manuscript file. These comments, excluding minor textual revisions, have been copied into this Peer Review File.

**Reviewer comments & decisions:**

**Reviewer comments, first version:**

Reviewers' comments:

Reviewer #1 (Remarks to the Author):

CENP-A is an epigenetically inherited histone that marks the centromere. CENP-A nucleosomes are remarkably stable. However, during replication and transcription, DNA- and RNA- polymerases must disassemble nucleosomes in order to progress along the chromatin. It has been shown that CENP-A nucleosomes are stably maintained during these cellular processes. However, it is currently unclear how CENP-A is maintained during transcription of centromeric repeats. For Canonical H3 nucleosomes that disassemble ahead of a progressing RNA-polymerase II (RNAPII) the histone chaperone called FACT which acts to promote disassembly and reassembly of the nucleosomes following the passage of RNAPII together with the transcription elongation factor Spt6.

In this manuscript, the authors sue the Drosophila S2 cell system to demonstrate that Spt6 recycles expelled dCENP-A during centromeric transcription. The authors propose that Spt6 centromeric localization is cell cycle regulated during mitosis and G1 phases, which are the cell cycle stages where centromere-associated transcripts and centromeric RNAPII have been previously identified, and when new CENP-A deposition is occurring. Furthermore, they show that degradation of Spt6 leads slight reduction of total CENP-A at the centromeres. Using the RITE system coupled with a degradable SPT6, the authors show that levels of existing CENP-A nucleosomes are significantly reduced at the centromere after Spt6 depletion. The work demonstrates a direct interaction between CENP-A and Spt6 and suggest that binding is affected by the phosphorylation state of CENP-A. A lack of phosphorylation strengthens the interaction, which correlates with increased centromeric signals of the phospho-defective mutant of CENP-A. The authors also conduct siRNA experiments in HeLa cells to show that the CENP-A maintenance defect in response to Spt6 depletion also occurs in human cells.

Overall, the major claim of the manuscript is novel, and helps provide a substantial piece of the CENP-A maintenance puzzle. The quality of the experimental work is high, and provides a convincing argument for a role for Spt6 in retention of dCENP-A. However, there are several issues listed below that should be addressed prior to acceptance of the manuscript.

Major Issues
1. The authors propose that the RITE system does not fully recapitulate the effects of Spt6 suppression on steady-state dCENP-A levels because the level of new CENP-A produced (dCENP-A-Myc) is not sufficient. The authors should provide western blot analysis to demonstrate that this is the case.
2. The authors use a 4-OHT inducible system to show that when CENP-A is overexpressed, increased new CENP-A deposition can occur in the Spt6 suppressed condition. This effect could also be due to an effect of Spt6 on expression of the 4OHT construct. The authors should demonstrate whether there is a similar amount of available dCENP-A in the Spt6 +/- conditions by western blot and RT-PCR.
3. The analysis of the phosphomutants is extremely interesting, but the effect of the dCENP-A S77A is not completely consistent with the general model put forward by the authors, and the field in general. The authors suggest that S77A enhances the ability of CENP-A to interact with Spt6. However, if CENP-A is completely stable under conditions where Spt6 is active/available, how does the enhanced interaction lead to MORE dCENP-A at centromere. An immunoblot should be done to preclude the possibility that there a difference in stability of wild-type dCENP-A versus the S77A mutant. If there is no difference, the authors need to discuss where this additional pool of CENP-A is derived. For example, is there a low level of dCENP-A loss that has not been measured previously?
4. The data provided showing that Spt6 depletion has a similar effect in human cells is interesting, and enhances the work. However, the level of analysis of the human phenotype types is very limited compared with the work in drosophila cells, and cannot preclude indirect effects on transcriptional levels of genes involved in CENP-A maintenance, such as CENP-C and HJURP. This caveat should be clearly stated in the text.

Minor Issues
1. Abbreviations used without definition: FACS, IF, co-IP.
2. Error bars are needed in Figure 1c.
3. The authors should provide the percent dCENP-A loss in figure 2d.
4. Also in figure 2d, notation for different levels of significance are provided in the figure legend, but only the p<0.05 is used in the figure.
5. In Supp Figure 2c, images of mitotic defects as a result of RNAi for Spt6 and white are shown, but not for dCENP-A.
6. Since the RITE system is new here, it would be good to provide validation that the new and old populations act as predicted, i.e. new is only loaded in M/G1.
7. Higher resolution images of human CENP-A staining should be displayed in figure 4, as is done for dCENP-A in figure 1.


Reviewer #2 (Remarks to the Author):

As CENP-A is a critical epigenetic marker for centromere specification, it is an important question how CENP-A is retained in centromere region during cell cycle progression. Authors in this MS have previously shown that centromeric transcription is required for CENP-A incorporation in Drosophila centromeres. In this study, they demonstrated that histone chaperon and transcription elongation factor Spt6 prevents the loss of old CENP-A. In addition, they showed that unphosphorylated CENP-A preferentially binds to Spt6. In total, authors proposed that Spt6 acts as a conserved CENP-A maintenance factor.
Although I agree that Spt6 plays something in CENP-A chromatin, current data do not completely support with authors’ conclusion. In authors’ model, Spt6 selectively retains old CENP-A, but not H3. But, I think that both H3 and CENP-A binds to Spt6. Although they mentioned that CENP-A-Spt6 binding is much stronger than H3-Spt under high salt condition, data are not clear, and authors need more detail analyses for this conclusion. In addition to this main point, I have several concerns authors should address before publication of this MS.

Specific points are
1. Localization analyses for Spt6 were not clear in Figure 1. High quality data are necessary.
2. RNAi depletion analyses are not necessary. Authors made a cell line, in which Spt6 can be degraded rapidly. They should perform phenotype analyses using this line, instead of RNAi analyses (Supplementary Fig. 2).
3. In Figure 2 authors demonstrated that total CENP-A levels were reduced after Spt6 depletion. It may be better to show immunoblot data for CENP-A before and after Sp6 depletion.
4. Experiments for human Spt6 are hard for interpretation. Partial depletion is not good experiment, because it is unclear what they observed. I strongly recommend that they should remove these data.
5. As mentioned above, biochemical analyses for Spt6-CENP-A or Spt6-H3 are unclear. In Figure 5b, why H4 band is faint? H4 should be same amount of H3. Bands of IP experiments are a little unclear. It is hard to conclude that CENP-A is better binding to Spt6 than H3 with current IP data. They should improve entire quality of biochemical experiments.
6. I am curious to know timing of CENP-A phosphorylation (S20/S75/S77). If authors can monitor timing of the phosphorylation (using phospho-antibodies), it would improve quality of the paper.
7. For Figure 6 experiments, author should perform CENP-A incorporation and retention activity assay, as they did in Figure 3.
8. Although the model authors proposed is interesting, it is still unclear how Spt6 functions for old CENP-A retention. If authors model is correct, CENP-A should be dynamic. If authors can show additional evidences to support their model, it would be good.


Reviewer #3 (Remarks to the Author):

Title: Spt6 is a maintenance factor for centromeric CENP-A
Authors: Bobkov et al.

The deposition of CENP-A is an important area of biology and this manuscript describes a new role for a histone chaperone and transcription elongation factor Spt6 in maintenance of old CENP-A nucleosomes in both Drosophila and human cells, which coincides with centromeric transcription. Authors have used cell biology based assays to show co-localization of Spt6 with CENP-A and provide evidence for an interaction with dCENP-A in vivo and in vitro. They report an increased interaction of Spt6 with phosphorylation deficient dCENP-A mutants than with histone H3. The authors have used multiple approaches to deplete Spt6 and also monitor new versus old CENP-A for their studies and optimized the protocols as best as they could. The authors have done studies with human cells to show that the role of Spt6 may be conserved in both flies and human cells. Whilst the data are suggestive of a role of Spt6 in deposition of CENP-A the observed defects may be due to direct/indirect effect of transcription inhibition both at the centromeres and globally. There are several issues that should be addressed as outlined below.

1) For Figure 1, the conclusion is that Spt6 prominently stains euchromatic regions. Is it fair to distinguish euchromatin from heterochromatin simply with DAPI staining? A better phrasing may be non-centromeric.
2) Spt6 associates with centromeric chromatin in G1 and mitotic cells. However, the reasons for its mitotic interaction with centromeres are unclear. Is there a role for mitotic association of Spt6 in not only regulating CENP-A but other aspects of centromere function such as transcription of centromeres?
3) Cell biology based assays have been used for localization and signal intensities quantified. Why haven’t the authors used ChIP experiments to show association of Spt6 with centromeres in Drosophila and human cells?
4) Phosphorylation of CENP-A is proposed as a mechanism by which Spt6 regulates CENP-A at the centromeres. There are several issues with this conclusion for example: a) Decreased intensities of dCENP-A-S77D at the kinetochore were observed, whereas increased intensities were observed for dCENP-A-S77A. There is no direct evidence that this phenotype is caused by Spt6. The non-phosphorylatable dCENP-A-S77A showed better interaction with Spt6 but this may not contribute to the observed localization of the the mutant CENP-A proteins. The differences in intensities could be independent of phosphorylation, and possibly caused by structural defects in the protein. b) The western blots in Figures 5C and 5D are not acceptable as they have several different exposures which have been spliced together. Levels of both IP dCENP-A and H3 (150mM NaCl) are different between WT and mutant (Figure 5D). Quantification of replicates is required is required with equal loading of the input.
5) The degron system was used to deplete Spt6 in Figure 2. The signal for dCENP-A signals were clearly reduced in Spt6-depleted cells based on signal intensity (Fig 2c). I could also see many dCENP-A foci in Spt6-depleted cells (see original figures for Shield1). There might be biological effects for Spt6-depleted cells since you can see similar dCENP-A foci in Spt6-depleted cells shown in Fig 3e and 3h. What are they? Can another marker be used for centromeres?
6) Why not include the data for the cyclin B in supplemental?
7) For the conclusion that Spt6 is required for transcription-mediated chromatin remodeling, the authors have used two approaches: a) centromere-associated transcripts associated with Spt6 (Figure 1F) and b) incorporation of old vs new CENP-A at the centromere. In their recent paper (Bobkov 2018) the authors have used localization of RNA PolII, PCNA, RNA-FISH, transcription inhibition to show a role for centromeric transcription. Hence, to strengthen their conclusion, the authors should consider doing the following: a) a transient inhibition of transcription to see if levels of centromeric Spt6 are altered, b) if levels of centromeric RNA and RNA PolII are affected upon depletion of Spt6 and c) co-localizaiton of Spt6 with RNA PolII.
8) Supplemental Figure 2. Authors compared the mitotic defect phenotype for Spt6 RNAi-2 day with CENP-A depletion-7 days. Why was day7 depletion used for for CENP-A? Western blots have not been included to show the depletion efficiency of CENP-A on different days. The reduced cell number in Spt6 RNAi suggests less proliferation, However, FACS analysis did not show any biased accumulation of cells in G1 or S phase in Spt6 RNAi compared to control RNAi. It would be of interest to quantify viable cells vs dead cells in Spt6 RNAi condition.
9) Pronounced reduction in old CENP-A, without much change in total CENP-A, indicates partial compensation by increased incorporation of new CENP-A. This is shown in Fig 3C, although the p-values are not significant. Authors further used deGradFP system and showed that there was significantly increased incorporation of new CENP-A. Why is there discrepancy between results in Fig 3C and Fig 3G.
10) Does depletion of Spt6 affect the transcription of CENP-A in the experimental set us used?
11) Studies from budding and fission yeast have proposed a role for FACT in preventing mislocalization of CENP-A. Is this true in flies and human cells? Do the authors see ectopic localization of CENP-A? Is CENP-A more stable after depletion of Spt6?
12) Other English edits: Paragraph one of the introduction (pg 2): “While canonical H3 is replenished during DNA replication in S-phase (citations), loading of CENP-A in Drosophila, humans as well as other vertebrates and takes place in a replication-dependent manner from late mitosis to G1.” The “and” after vertebrates should not be present and it would read better as: While canonical H3 is replenished during DNA replication in S-phase (citations), loading of CENP-A in Drosophila, humans, and other vertebrates takes place in a replication-dependent manner from late mitosis to G1. In the last paragraph of the discussion (pg 13), organism should be plural.

**Author rebuttal, first version:**

Reviewers' comments:

Reviewer #1 (Remarks to the Author):

CENP-A is an epigenetically inherited histone that marks the centromere. CENP-A nucleosomes are remarkably stable. However, during replication and transcription, DNA- and RNA- polymerases must disassemble nucleosomes in order to progress along the chromatin. It has been shown that CENP-A nucleosomes are stably maintained during these cellular processes. However, it is currently unclear how CENP-A is maintained during transcription of centromeric repeats. For Canonical H3 nucleosomes that disassemble ahead of a progressing RNA-polymerase II (RNAPII) the histone chaperone called FACT which acts to promote disassembly and reassembly of the nucleosomes following the passage of RNAPII together with the transcription elongation factor Spt6.

In this manuscript, the authors sue the Drosophila S2 cell system to demonstrate that Spt6 recycles expelled dCENP-A during centromeric transcription. The authors propose that Spt6 centromeric localization is cell cycle regulated during mitosis and G1 phases, which are the cell cycle stages where centromere-associated transcripts and centromeric RNAPII have been previously identified, and when new CENP-A deposition is occurring. Furthermore, they show that degradation of Spt6 leads slight reduction of total CENP-A at the centromeres. Using the RITE system coupled with a degradable SPT6, the authors show that levels of existing CENP-A nucleosomes are significantly reduced at the centromere after Spt6 depletion. The work

demonstrates a direct interaction between CENP-A and Spt6 and suggest that binding is affected by the phosphorylation state of CENP-A. A lack of phosphorylation strengthens the interaction, which correlates with increased centromeric signals of the phospho-defective

mutant of CENP-A. The authors also conduct siRNA experiments in HeLa cells to show that the CENP-A maintenance defect in response to Spt6 depletion also occurs in human cells.

Overall, the major claim of the manuscript is novel, and helps provide a substantial piece of the CENP-A maintenance puzzle. The quality of the experimental work is high, and provides a convincing argument for a role for Spt6 in retention of dCENP-A. However, there are several issues listed below that should be addressed prior to acceptance of the manuscript.

We would like to thank the reviewer for acknowledging the high quality of our experimental work. This is very much appreciated. Please find our answers to the issues raised below:

Major Issues

1. The authors propose that the RITE system does not fully recapitulate the effects of Spt6 suppression on steady-state dCENP-A levels because the level of new CENP-A produced (dCENP-A-Myc) is not sufficient. The authors should provide western blot analysis to demonstrate that this is the case.

We agree that this is an important experiment and required to support our hypothesis that the tamoxifen system produces more “new dCENP-A” than the RITE system and thus allows more incorporation of new dCENP-A (TI-dCENP-A^HA^ = dCENP-A-HA-ERT2) as compared to new CENP-A provided by the RITE system. As suggested by the reviewer, we have now performed Western blots using anti-dCENP-A antibody to analyse the levels of the transgenic dCENP-A constructs relative to wildtype (new Supplementary fig. S3c and d) with an explanation in the main manuscript.

However, it is not possible to assess directly the levels of the “exchanged” new myc-tagged protein by western blotting in the RITE system in a meaningful way. This is due to technical reasons as the Cre-recombinase treatment only induces an exchange of the tag from v5 to myc in a subset of cells (about ≈20-40%; presumably not all cells take up the recombinant Cre-Recombinase). While it is easy to recognise and quantify the cells that underwent successful tag-exchange using microscopy, a population-based assay like a Western blot does not give an accurate reading of average dCENP-A-myc levels. However, the expression levels of the promoter for both the dCENP-A-v5 and the dCENP-A myc should remain the same before and after the tag-exchange. Thus, the levels of the transgenic dCENP-A-v5 relative to endogenous dCENP-A can be used as a proxy for the levels of the recombination-switched new dCENP-A-myc. Using a general anti-dCENP-A antibody, we are able to distinguish the higher molecular weight transgene product (dCENP-A-V5) by size from endogenous dCENP-A as a reference point.

Also note that expression levels of the endogenous CENP-A adapt to lower levels in the presence of a constitutively expressed transgene as reported in

human cells (Foltz et al., NCB 2006, Fig. S1a, b). We observe this also in Drosophila cells (Supplementary Fig. 3c), hence a stronger band of dCENP-A can be seen for the wildtype control.

Performing the Western blots as described, we find that the level of the transgenic dCENP-A-V5 product in the RITE system is similar to the endogenous dCENP-A levels (Supplementary figure 3c; -Shield1) and much lower than the transgenic TI-dCENP-A^HA^ product of the Tamoxifen system (Supplementary figure 3d). This result supports our interpretation that levels of the transgene in the RITE system (e.g. dCENP-A-myc), although allowing incorporation of some new dCENP-A +/- Spt6, are too low to contribute to the enriched incorporation of new dCENP-A as observed in the Tamoxifen system. We also observed a reduction in total endogenous dCENP-A levels upon Spt6 depletion in line with the microscopy analysis of total dCENP-A at centromeres (Fig. 2d). However, this result should be taken with caution, because although, western blotting is a good read-out of all CENP-A in the chromatin, both centromeric and non-centromeric pools of CENP-A contribute to it. It was estimated in human cells that the majority of CENP-A (≈ up to 66%) is in the non-centromeric genome (Bodor et al., 2014). For this reason, we think that the image analysis focused on centromeric dCENP-A incorporation is more meaningful for this study and decided to add the western blots as supplementary data.

2. The authors use a 4-OHT inducible system to show that when CENP-A is overexpressed, increased new CENP-A deposition can occur in the Spt6 suppressed condition. This effect could also be due to an effect of Spt6 on expression of the 4OHT construct. The authors should demonstrate whether there is a similar amount of available dCENP-A in the Spt6 +/- conditions by western blot and RT-PCR.

In principle, we would not expect an effect on the transgenic TI-dCENP-A^HA^ construct in the tamoxifen system, since the original purpose of this system was to provide ready-made “new” dCENP-A without relying on new transcription. Still, we performed the requested Western blots in the tamoxifen-inducible system and as expected found that the +/- Spt6 conditions had no noticeable impact on the levels of the Tamoxifen construct TI-dCENP-A^HA^ (Supplementary figure 3d).

3. The analysis of the phosphomutants is extremely interesting, but the effect of the dCENP-A S77A is not completely consistent with the general model put forward by the authors, and the field in general. The authors suggest that S77A enhances the ability of CENP-A to interact with Spt6. However, if CENP-A is completely stable under conditions where Spt6 is active/available, how does the enhanced interaction lead to MORE dCENP-A at centromere. An immunoblot should be done to preclude the possibility that there a difference in stability of wild-type dCENP-A versus the S77A mutant. If there is no difference, the authors need to discuss where this additional pool of CENP-A is derived. For example, is there a low level of dCENP-A loss that has not been measured previously?

The reviewer raises a very interesting question that we hadn’t addressed in the previous study. Indeed, if dCENP-A is completely stable, how is it possible that the S77A accumulates relative to wildtype?

In order to answer this question, we have performed a time course experiment using a pulse-chase approach and followed the decline of newly incorporated versions of dCENP-A (WT, S77A and S77D) in mitotic cells (new Fig 6 e-h and Supplementary Fig. 5). Mitotic cells offer the advantage of quantifying individual centromeres as opposed to interphase centromere clusters.

Unexpectedly, we found evidence for this reviewer’s assumption, namely that dCENP-A is not a 100% stable apart from replicative dilution. In fact, we uncovered a low level of dCENP-A loss, in agreement with recent findings by the Cheeseman lab (Swartz et al., Dev Cell 2019). We observe a less than perfect inheritance for wildtype dCENP-A (20% instead of 25%) after two cycles (new Fig 6f and g). In contrast, the S77A mutant is much closer to perfect inheritance (23%) while the phosphomimetic mutant is much lower at 13%. We also calculated the decay rate constant for each mutant of at 48h after pulse labelling and found them to be significantly different from each other (new Fig 6h). This result is an important finding as it supports the notion that CENP-A is indeed not completely stable. It also now provides an explanation for how it is possible that the S77A mutant can accumulate relative to wildtype dCENP-A.

4. The data provided showing that Spt6 depletion has a similar effect in human cells is interesting, and enhances the work. However, the level of analysis of the human phenotype types is very limited compared with the work in drosophila cells, and cannot preclude indirect effects on transcriptional levels of genes involved in CENP-A maintenance, such as CENP-C and HJURP. This caveat should be clearly stated in the text.

We completely agree with the reviewer that this data is an important piece in this study extending its significance across species boundaries. We have now extended our analysis of Spt6 depletion in human cells. We now used two different siRNAs to partially deplete Spt6 that resulted in a similar defect, a reproducible 20% reduction in CENP-A maintenance for a single cell cycle (see new Fig 4b and d). We also controlled for proper transition of the cells into G1, by demonstrating lack of Cyclin B staining after the thymidine arrest (new Supplementary Fig 4a and b). As in the previous manuscript, we used depletion of CENP-C as a positive control to monitor lack of CENP-A maintenance (about 50% reduction). We now show that CENP-C levels are not changed during SPT6 depletion in either siRNA probe indicating that loss of old CENP-A is not an indirect consequence of CENP-C loss (new Fig 4b).

Minor Issues

1. Abbreviations used without definition: FACS, IF, co-IP.

Done

2. Error bars are needed in Figure 1c.
   Done
3. The authors should provide the percent dCENP-A loss in figure 2d. Percentages for dCENP-A loss are now provided in the text for both Figure 2d (17%) and 3d (58%).
4. Also in figure 2d, notation for different levels of significance are provided in the figure legend, but only the p<0.05 is used in the figure.

Sorry this was an oversight on our part from a previous arrangement of panels in Figure 2 and is now corrected.

5. In Supp Figure 2c, images of mitotic defects as a result of RNAi for Spt6 and white are shown, but not for dCENP-A.

An image for the mitotic defect in dCENP-A RNAi cells is now added to the figure, with a brief description in the main text. As reported previously (Blower et al., 2001, Heun et al.,2006) many dCENP-A RNAi cells display chromosomes with aberrant prometaphase congression. In these cells the chromosomes can be observed spread out alongside the mitotic spindle, with the bulk of the chromosome mass close to the cell equator.

6. Since the RITE system is new here, it would be good to provide validation that the new and old populations act as predicted, i.e. new is only loaded in M/G1. Unfortunately, the technical setup of the RITE system does not allow for high temporal resolution (<< one generation time) to determine the cell cycle stage of dCENP-A loading. This is due to two reasons: 1) Currently, Schneider S2 cells cannot be synchronised and 2) the time required to induce a detectable recombination switch (21h) is just short of one generation time (about 24 h). Incorporation of CENP-A in different organisms is regulated at the protein level. Moreover, several labs (Lehner, Karpen) including our own have previously demonstrated in Drosophila that transgenic dCENP-A constructs are incorporated in M/G1 independent of their tag (Lidsky et al., 2011, Dunleavy et al., 2012, Bobkov et al., 2018). In Bobkov et al., we already used the MT-promoter (low level, non-induced metallothionein-promoter) as in the RITE system and two different versions of dCENP-A, namely TI-dCENP-A^HA^ (also used in this study) and dCENP-A^mCherr^y. All of these constructs incorporate in the correct time window of the cell cycle. Taken together, we think it is highly unlikely that dCENP-A^V5^ and dCENP-A^MYC^ should behave any differently when expressed from the same MT-promoter.
7. Higher resolution images of human CENP-A staining should be displayed in figure 4, as is done for dCENP-A in figure 1.

Higher resolution images with a narrower field of view are now displayed in Figure 4c.

Reviewer #2 (Remarks to the Author):

As CENP-A is a critical epigenetic marker for centromere specification, it is an important question how CENP-A is retained in centromere region during cell cycle progression. Authors in this MS have previously shown that centromeric transcription is required for CENP-A incorporation in Drosophila centromeres. In this study, they demonstrated that histone chaperon and transcription elongation factor Spt6 prevents the loss of old CENP-A. In addition, they showed that unphosphorylated CENP-A preferentially binds to Spt6. In total, authors proposed that Spt6 acts as a conserved CENP-A maintenance factor.

Although I agree that Spt6 plays something in CENP-A chromatin, current data do not completely support with authors’ conclusion. In authors’ model, Spt6 selectively retains old CENP-A, but not H3. But, I think that both H3 and CENP-A binds to Spt6. Although they mentioned that CENP-A-Spt6 binding is much stronger than H3-Spt under high salt condition, data are not clear, and authors need more detail analyses for this conclusion. In addition to this main point, I have several concerns authors should address before publication of this MS.

We thank the reviewer for his critical input but we think the interesting question that the reviewer raised, is beyond the scope of this paper. The reviewer is correct that Spt6 was initially shown to bind directly H3/H4 tetramers (in budding yeast). We have confirmed this for Drosophila (Figure 5) and in addition demonstrate that it also binds directly to CENP-A/H4 (Figure 5). It is therefore likely that Spt6 binds to all H3 variants. However, the exact mode of binding even to canonical H3/H4 is a matter of ongoing investigation which would be a prerequisite to investigate how Spt6 can distinguish between different variants of H3. While our data supports a model that recognition of CENP-A involves phospho-regulation how this changes affinities and distinction from H3 is still unclear and will be a major research focus of us in the future.

Specific points are

1. Localization analyses for Spt6 were not clear in Figure 1. High quality data are necessary.

Spt6 is an abundant protein and as a transcription elongation factor, it is expected to strongly stain large euchromatic regions in interphase. Likely its abundance also causes substantial background staining in mitosis, where Spt6 is largely displaced from chromosomes and found in the cytoplasm. Despite this, we find clear evidence of Spt6 localisation at the centromere, both using anti-Spt6 antibody and a transgenic Spt6-GFP construct. Although the signal of Spt6 signal is weaker at centromeres as compared to the euchromatin stain, it generally follows the pattern of dCENP-A and labels most centromeres. To illustrate this better we have now added yellow arrows to point out Spt6 labelled centromeres. We also agree that the metaphase in Figure 1b could have shown a more convincing inset on a different centromere. This is now changed in the new Figure 1. To further illustrate that Spt6 localises at the centromere, we have now also added a panel of additional images in the Supplementary Figure 1a. This image features a mitotic chromosome spread, which shows co-localisation of endogenous Spt6 with centromeres on individualised chromosomes.

2. RNAi depletion analyses are not necessary. Authors made a cell line, in which

Spt6 can be degraded rapidly. They should perform phenotype analyses using this line, instead of RNAi analyses (Supplementary Fig. 2).

We have included the RNAi depletion as supplemental data because the mitotic defect phenotype was one of the criteria (in addition to centromere staining) that led us to focus on Spt6 out of a large number of candidate genes from Barth, Bobkov et al., 2014). Using the adapted Shield1-deGradFP system we did not notice an increase in mitotic defects, likely due to the short time period of 21h during which the Spt6 depletion took place. This information is now added to the manuscript. Note that we purposely kept the time less than one generation (about 24h) to avoid secondary effects such as the cell cycle block observed in the RNAi. As stated previously, maintaining the Spt6 depleted cells beyond one generation resulted in growth arrest.

3. In Figure 2 authors demonstrated that total CENP-A levels were reduced after Spt6 depletion. It may be better to show immunoblot data for CENP-A before and after Spt6 depletion.

The depletion of total dCENP-A shown in Figure 2d amounts to a reduction of 17%. We have performed the western blot for dCENP-A before and after Spt6 depletion and find some reduction of the band for the endogenous dCENP-A yet not an obvious change for the dCENP-A-V5 transgenic construct. As pointed out in the answer to the first question of reviewer 1, our view is that microscopy quantification is better suited to specifically quantify CENP-A levels as compared to immunoblotting. The western blot reads out total levels of dCENP-A in the genome, rather than concentrating on the centromeric CENP-A pool. Quantification in human cells showed that the majority of CENP-A (≈60-70%) is in the non-centromeric genome (Bodor et al., 2014), but is much more diluted and therefore below the level of detection in microscopy. We expect this to be similar in Drosophila as non-centromeric localisation of dCENP-A can be visualised in overexpression settings (seen for both human and Drosophila cells; Heun et al. 2005, Olszak et al., 2011, Gascoigne et al., 2011). Taken together, we think that immunoblotting data is not the correct way to assess the levels of centromeric CENP-A due to the aforementioned technical issues. We clearly failed to clarify this point sufficiently initially as two reviewers suggested the same experiment. We have now expanded our reasoning for this in the new manuscript.

4. Experiments for human Spt6 are hard for interpretation. Partial depletion is not good experiment, because it is unclear what they observed. I strongly recommend that they should remove these data.

We agree that partial depletions are in principle more difficult to interpret. However, in the case of essential genes, reduced expression offers an opportunity to identify a dose dependent role, without causing major secondary effects (e.g. cell death). In the case of Spt6, partial depletion is necessary to avoid the cell cycle block, which would prevent CENP-A assembly, which is itself cell cycle coupled. We agree with the reviewer that the Spt6 depletion experiments in human cells required a more detailed analysis. This we have now done by: 1) addition of a second independent siRNA construct resulting in the same reduction in centromeric CENP-A levels (Figure 4b-d), 2) control for CENP-C levels following Spt6 depletion as CENP-C is a well-known factor stabilizing CENP-A (Figure 4b) and 3) ensure complete cell cycle progression into G1

(absence Cyclin B staining in the vast majority of cells; Supplementary Figure 4a and b).

While bringing the human cell experiments up to the same level of rigor and engineering as the Drosophila experiments is beyond the scope of this paper, we strongly feel that the demonstration of a conserved role of Spt6 on CENP-A dynamics is a valuable addition to this work. It extends our results from a mere Drosophila-specific phenomenon to a common mechanism that is utilized in human cells as well. We hope that the new data provided in figure 4 convinces reviewer 2 of our decision to include it in the revised manuscript.

5. As mentioned above, biochemical analyses for Spt6-CENP-A or Spt6-H3 are unclear. In Figure 5b, why H4 band is faint? H4 should be same amount of H3. Bands of IP experiments are a little unclear. It is hard to conclude that CENP-A is better binding to Spt6 than H3 with current IP data. They should improve entire quality of biochemical experiments.

We agree with the reviewer that the H4 band is weaker than the corresponding H3 or the dCENP-A-ΔNT band although it is expected to be similarly stained by Coomassie. Independently, the biochemical analysis is still clear, we see recombinant CENP-A and H3 in a Spt6-fragment pulldown, which is absent in the beads control. We do not know why the band is weaker, but in previous repeats of this experiment we obtained the same result. We now commented on this observation in the manuscript, speculating that Spt6 also binds a small population of H3 or dCENP-A that is not associated with H4.

Regarding the IP experiment, we agree that some of the protein bands detected on the western blots could have been clearer. We have therefore repeated all the IPs in Figure 5c and d and obtained this time stronger signals, which are now shown in the new Figure 5.

As shown previously with the weaker signals we find that the phosphomimetic aspartate-mutant associates less well with Spt6 as compared to the WT and non-phos. alanine-mutants, with the triple-alanine mutant showing the strongest IP-signal.

In Figure 5e it is now much clearer that wildtype dCENP-A shows more specific binding to Spt6 at high-salt concentrations (750 mM) than H3. In this new set of experiments, we only observed a small to no detectable difference between WT and the alanine mutant, as both interacted well with Spt6 at 750 mM. We have now changed the description of this experiment accordingly in the manuscript.

6. I am curious to know timing of CENP-A phosphorylation (S20/S75/S77). If authors can monitor timing of the phosphorylation (using phospho-antibodies), it would improve quality of the paper.

The reviewer raises a very interesting question that we would very much like to investigate. Unfortunately, as currently no phospho-specific antibodies for these marks exist, these experiments need to be relegated to the future.

7. For Figure 6 experiments, author should perform CENP-A incorporation and retention activity assay, as they did in Figure 3.

To address this question, we have used the SNAP-tag approach in a pulse-chase experiment to test for incorporation and retention of dCENP-A quantifying single centromeres.

As outlined in our answer to reviewer 1 question 3, we have performed a time course experiment to follow the decline of newly incorporated versions of dCENP-A (WT, S77A and S77D) in mitotic cells (new Fig. 6 e-h and Supplementary Fig. 5). We decided to concentrate on mitotic cells as they offer the advantage of quantifying individual centromeres rather than interphase centromere clusters. Interestingly, we found evidence that dCENP-A is not a 100% stable (new Fig. 6 f, g). In fact, we uncovered a low level of dCENP-A loss (similar to the very recent findings of the Cheeseman lab; Swartz et al., Dev Cell 2019), which now provides an explanation for why an S77A mutant can accumulate relative to wildtype dCENP-A.

8. Although the model authors proposed is interesting, it is still unclear how Spt6 functions for old CENP-A retention. If authors model is correct, CENP-A should be dynamic. If authors can show additional evidences to support their model, it would be good.

Previous publications indicated that levels of old CENP-A are completely stable. Although CENP-A is indeed very stable, we and others (Swartz et al., Dev Cell 2019) now demonstrate that even in normal conditions not all dCENP-A is maintained (Figure 6; please see also the discussion of point 7). As suggested by the reviewer, one could argue that CENP-A shows a small level of dynamics, which is elevated in sensitized conditions such as phosphomimetic residues on CENP-A or Spt6 depletion.

Reviewer #3 (Remarks to the Author):

Title: Spt6 is a maintenance factor for centromeric CENP-A
Authors: Bobkov et al.

The deposition of CENP-A is an important area of biology and this manuscript describes a new role for a histone chaperone and transcription elongation factor Spt6 in maintenance of old CENP-A nucleosomes in both Drosophila and human cells, which coincides with centromeric transcription. Authors have used cell biology based assays to show co-localization of Spt6 with CENP-A and provide evidence for an interaction with dCENP-A in vivo and in vitro. They report an increased interaction of Spt6 with phosphorylation deficient dCENP-A mutants than with histone H3. The authors have used multiple approaches to deplete Spt6 and also monitor new versus old CENP-A for their studies and optimized the protocols as best as they could. The authors have done studies with human cells to show that the role of Spt6 may be conserved in both flies and human cells. Whilst the data are suggestive of a role of Spt6 in deposition of CENP-A the observed defects may be due to direct/indirect effect of transcription inhibition both at the centromeres and globally. There are several issues that should be addressed as outlined below.

We would like to thank this reviewer for his/her constructive criticism.

1) For Figure 1, the conclusion is that Spt6 prominently stains euchromatic regions. Is it fair to distinguish euchromatin from heterochromatin simply with DAPI staining? A better phrasing may be non-centromeric.

We agree with the reviewer that DAPI staining is not a good way to distinguish euchromatin from heterochromatin and did not intend to say so. The distinction between eu- and heterochromatin was made using Spt6 staining indicative of transcriptionally active regions. We have now changed the text according to the reviewers request to reflect this more clearly.

2) Spt6 associates with centromeric chromatin in G1 and mitotic cells. However, the reasons for its mitotic interaction with centromeres are unclear. Is there a role for mitotic association of Spt6 in not only regulating CENP-A but other aspects of centromere function such as transcription of centromeres?

Indeed, our model is that Spt6 can be found at the centromere because of active transcription at centromeric DNA. We have previously demonstrated the presence of RNA PolII and nascent transcripts in mitosis and G1 (Bobkov et al., JCB, 2018). Since Spt6 can directly associate with RNAPII through its SH2 domain, the most straightforward interpretation is that Spt6 gets targeted to centromeres via centromeric transcription, which can also be inferred from Figure 1f. During centromeric transcription, Spt6 exerts its important function, namely to specifically retain old CENP-A, which were displaced by transcription-coupled nucleosome eviction.

3) Cell biology based assays have been used for localization and signal intensities quantified. Why haven’t the authors used ChIP experiments to show association of Spt6 with centromeres in Drosophila and human cells?

This has to do with the fact that at the time of the experiment the highly repetitive centromeric DNA was not assembled for Drosophila. Only recently, Chang et al, 2019 managed to overcome the issue of repetitive DNA at centromeres and succeeded in a first assembly (Chang et al., PLoS Biology, 2019). We agree that it would be interesting to obtain a higher resolution view of Spt6 binding at the centromere, and we are planning to do this experiment in the future. For the claim that Spt6 colocalizes with dCENP-A at centromeres, we feel that our immune staining and live cell imaging experiments are well over the current bar to support such a claim.

4) Phosphorylation of CENP-A is proposed as a mechanism by which Spt6 regulates CENP-A at the centromeres. There are several issues with this conclusion for example: a) Decreased intensities of dCENP-A-S77D at the kinetochore were observed, whereas increased intensities were observed for dCENP-A-S77A. There is no direct evidence that this phenotype is caused by Spt6. The non-phosphorylatable dCENP-A-S77A showed better interaction with Spt6 but this may not contribute to the observed localization of the the mutant CENP-A proteins. The differences in intensities could be independent of phosphorylation, and possibly caused by structural defects in the protein. b) The western blots in Figures 5C and 5D are not acceptable as they have several different exposures which have been spliced together. Levels of both IP dCENP-A and H3 (150mM NaCl) are different between WT and mutant (Figure 5D).

Quantification of replicates is required is required with equal loading of the input.

Reviewer 3 is correct and we did not intend to claim that the data display in Figure 6 does provide direct evidence that the loss of the CENP-A-S77D mutant or the accumulation of the CENP-A-S77A mutant is linked to Spt6. Instead, we only investigated whether the behaviour of these mutants correlated with our expectation based on the Spt6-IP results. We do not think structural defects are likely, because these mutations are made in the unstructured N-terminal tail of CENP-A and far from the structured histone fold domain.

To address the concerns of this reviewer, we have conducted a much deeper analysis into the retention of these mutants by performing a time course experiment to follow the decline of newly incorporated versions of dCENP-A (WT, S77A and S77D) at individual centromeres on mitotic chromosomes (new Fig. 6, e-h and Supplementary Fig. 5).

Interestingly, we found evidence that dCENP-A is not a 100% stable and instead uncovered a low level of dCENP-A loss (new Fig. 6 f, g). This provides an explanation for why an S77A mutant can accumulate relative to wildtype dCENP-A. While it is indeed no direct evidence for involvement of Spt6 in the maintenance of these mutants, their behaviour agrees with our IP results.

b) We see the reviewers point and just to explain, it was not our intention to mislead the reader. The longer exposure for H3 was only shown to equalise the band intensity of H3 to CENP-A at 150 mM salt, to then better compare band intensities at 750 mM salt (the absolute intensities cannot really be compared, because they are obtained using different antibodies). Still, we generally agree with the reviewer that some of the IP’s simply produced too low signals, which weakened our interpretation of the results.

We have now repeated the IP experiments and western blots for 5c and 5d and obtained much stronger signals. As outlined in our answer to question 5 of reviewer 2, we were generally able to reproduce our previous findings, but with a much clearer data set. We find the phosphomimetic aspartate-mutant to associate less well with Spt6 as compared to the WT and single non-phos. alanine-mutants, whereas the triple-alanine mutant showed the strongest IP-signal. Interestingly, we see now much clearer that wildtype dCENP-A shows a more specific binding to Spt6 at high-salt concentrations (750 mM) than H3 (Fig 5d). As requested the loading has been adjusted to obtain equal input signal for the different experiments. In this new more convincing set of experiments we find that there is only a small to no detectable difference between WT and the alanine mutant, as both interact well with Spt6 at 750 mM. We have now changed the description of this experiment accordingly in the manuscript.

5) The degron system was used to deplete Spt6 in Figure 2. The signal for dCENP-A signals were clearly reduced in Spt6-depleted cells based on signal intensity (Fig 2c). I could also see many dCENP-A foci in Spt6-depleted cells (see original figures for Shield1). There might be biological effects for Spt6-depleted cells since you can see similar dCENP-A foci in Spt6-depleted cells shown in Fig 3e and 3h. What are they? Can another marker be used for centromeres?

We would like to thank the reviewer for pointing this out. It should be noted that S2 cells are pseudo-tetraploid (2xX, 4x 2^nd^, 4x 3^rd^ and 3x 4^th^). Even though they

normally cluster in about 3-5 foci and also occasionally show more foci representing individual centromeres. De-clustering of centromeres as a consequence of Spt6 removal would be indeed an interesting additional phenotype. However, when we analysed this further we did not find a significant difference between cells with or without Spt6.

6) Why not include the data for the cyclin B in supplemental? This is now shown in Supplementary Figure S4.
7) For the conclusion that Spt6 is required for transcription-mediated chromatin remodelling, the authors have used two approaches: a) centromere-associated transcripts associated with Spt6 (Figure 1F) and b) incorporation of old vs new CENP-A at the centromere. In their recent paper (Bobkov 2018) the authors have used localization of RNA PolII, PCNA, RNA-FISH, transcription inhibition to show a role for centromeric transcription. Hence, to strengthen their conclusion, the authors should consider doing the following: a) a transient inhibition of transcription to see if levels of centromeric Spt6 are altered, b) if levels of centromeric RNA and RNA PolII are affected upon depletion of Spt6 and c) co-localizaiton of Spt6 with RNA PolII.

We have now included additional data demonstrating co-localization of Spt6 with RNA PolII on mitotic chromosome spreads (Supplementary Fig. 1a). We also agree with the reviewer that the other mentioned experiments will be interesting to perform and are planning to investigate the relationship between Spt6, RNAPII and transcription inhibition at the centromere in more detail in the future. However, we think that this would unnecessarily overload our current story and a strong connection between Spt6 and transcription is already well described from budding yeast to human cells in numerous previous publications (for review Okazaki et al. Epigenetics 2013 and since then many primary papers including the recent Jeronimo C et al. in Cell Reports 2019.

8) Supplemental Figure 2. Authors compared the mitotic defect phenotype for Spt6 RNAi-2 day with CENP-A depletion-7 days. Why was day7 depletion used for for CENP-A? Western blots have not been included to show the depletion efficiency of CENP-A on different days. The reduced cell number in Spt6 RNAi suggests less proliferation, However, FACS analysis did not show any biased accumulation of cells in G1 or S phase in Spt6 RNAi compared to control RNAi. It would be of interest to quantify viable cells vs dead cells in Spt6 RNAi condition.

CENP-A in Drosophila and other organisms needs to be depleted for a certain amount of time before you can see defects. This has to do with the fact that cells appear to tolerate low amounts of CENP-A up to a reduction of 90%, before showing phenotypic effects. Therefore, RNAi takes time as previously made CENP-A is very stable and mostly depleted by replicative dilution (as also investigated in this study in the new Figure 6). This is now mentioned in the

text. When this study was undertaken, we monitored dCENP-A depletion after day 6 and 7 through immunofluorescence. A representative image of dCENP-A RNAi is now shown in Supplementary Figure 2 c to demonstrate depletion of the protein alongside with a typical phenotype, namely unaligned mitotic chromosomes.

It should be noted that dCENP-A RNAi is a well-established depletion experiment that has been performed multiple times in previous publications (Blower et al., 2001, Heun et al., 2006, Erhardt et al., 2008, Bobkov et al, 2014) leading to reproducible phenotypes. The experiment could be taken out if reviewer 3 insists, but we thought it makes sense to keep it for comparison with Spt6 regarding the severity of the phenotype and justify why we chose to focus on Spt6.

9) Pronounced reduction in old CENP-A, without much change in total CENP-A, indicates partial compensation by increased incorporation of new CENP-A. This is shown in Fig 3C, although the p-values are not significant. Authors further used deGradFP system and showed that there was significantly increased incorporation of new CENP-A. Why is there discrepancy between results in Fig 3C and Fig 3G.

We hypothesised in the previous manuscript that the observed overloading of new CENP-A (Fig 3g) comes from potentially higher protein levels of new CENP-A in the Tamoxifen system (TI-dCENP-A^HA^; Fig 3g) as compared to the RITE system (dCENP-A^MYC^; Fig 3c). We have now performed Western blots in both systems to assess endogenous and transgenic dCENP-A levels (new Supplementary Fig. 3c and d). Indeed, we observe that the level of the transgenic dCENP-A construct (TI-dCENP-A^HA^) is higher than the transgenic dCENP-A construct in the RITE system. This lends support to our interpretation that upon providing more “new dCENP-A” also more gets the opportunity to become incorporated in the Spt6 depletion conditions to fill the gaps left behind by old dCENP-A.

10) Does depletion of Spt6 affect the transcription of CENP-A in the experimental set us used?

Our data suggests that this is very unlikely for the following reason: Production of new CENP-A-myc in the RITE system depends on *de novo* transcription after Cre-Recombinase treatment. Importantly, in Spt6 depleted cells we only see a change in centromere signal for old dCENP-A-V5 but not for new CENP-A myc, which shows a trend for more loading albeit not significant. We have now changed the text to illustrate this point better.

11) Studies from budding and fission yeast have proposed a role for FACT in preventing mislocalization of CENP-A. Is this true in flies and human cells? Do the authors see ectopic localization of CENP-A? Is CENP-A more stable after depletion of Spt6?

No, we have not detected any evidence of mislocalisation of dCENP-A either in human or Drosophila cells, possibly due to the short time frame of the experiment.

12) Other English edits: Paragraph one of the introduction (pg 2): “While canonical H3 is replenished during DNA replication in S-phase (citations), loading of CENP-A in Drosophila, humans as well as other vertebrates and takes place in a replication-dependent manner from late mitosis to G1.” The “and” after vertebrates should not be present and it would read better as: While canonical H3 is replenished during DNA replication in S-phase (citations), loading of CENP-A in Drosophila, humans, and other vertebrates takes place in a replication-dependent manner from late mitosis to G1. In the last paragraph of the discussion (pg 13), organism should be plural.

We would like to thank the reviewer for spotting these mistakes. They are both now corrected as suggested.

**Reviewer comments, second version:**

REVIEWERS' COMMENTS:

Reviewer #1 (Remarks to the Author):

The authors have adequately addressed the critiques from the previous round of review. I have no further comments and support publication of the manuscript in Nat. Communications.


Reviewer #2 (Remarks to the Author):

I found that Bobkov et al. did additional works to address concerns raised by all Reviewers. Manuscripts were substantially improved and quality of the MS is much better. However, I have still concerns on data of partial RNAi experiments using human cells (Figure 4). In previous reviews, I recommended removal or improvements of these data. Although authors mentioned some reasons why they still wanted to use “partial” RNAi, I disagree with their explanation. If they wanted to use similar analysis using human cells, they must generate a conditional knockout human cell line. In my experiences, it is hard to control depletion level of a target protein using RNAi, and I am not convinced with such data. I never said that author should make a conditional knockout cell line, and recommend to remove human data. Although I understand that they want to say functional conservation of Spt6, it is not easy to say such a conclusion. If they want to say functional conservation with human cells, they need additional data (localization, biochemical and cell biological data). It may be better to prepare another paper using human cells. I believe that characterization of dSpt6 related with dCENP-A is really good work.


Reviewer #3 (Remarks to the Author):

The authors have addressed all of my concerns and done an excellent job on the revisions with new experimental data. I was also impressed with their response to other two reviewers. The paper is a significant contribution to the field.

**Author rebuttal, second version:**

Reviewer #1 (Remarks to the Author):

The authors have adequately addressed the critiques from the previous round of review. I have no further comments and support publication of the manuscript in Nat. Communications.


Reviewer #2 (Remarks to the Author):

I found that Bobkov et al. did additional works to address concerns raised by all Reviewers. Manuscripts were substantially improved and quality of the MS is much better. However, I have still concerns on data of partial RNAi experiments using human cells (Figure 4). In previous reviews, I recommended removal or improvements of these data. Although authors mentioned some reasons why they still wanted to use “partial” RNAi, I disagree with their explanation. If they wanted to use similar analysis using human cells, they must generate a conditional knockout human cell line. In my experiences, it is hard to control depletion level of a target protein using RNAi, and I am not convinced with such data. I never said that author should make a conditional knockout cell line, and recommend to remove human data. Although I understand that they want to say functional conservation of Spt6, it is not easy to say such a conclusion. If they want to say functional conservation with human
cells, they need additional data (localization, biochemical and cell biological data). It may be better to prepare another paper using human cells. I believe that characterization of dSpt6 related with dCENP-A is really good work.

This issue was discussed with the editor, who agreed that the manuscript benefits from the human data and the other two reviewers appear to think likewise. In order to emphasize more the early and more speculative stage of the research conducted into human Spt6, the editor suggested the following: 1) Make the human experiments to the last data figure (Fig. 6) closer to the discussion to emphasize its more speculative nature at this point. 2) Change the text and built in qualifiers to illustrate this point further. I have followed both recommendations, which can be seen in the updated manuscript.


Reviewer #3 (Remarks to the Author):

The authors have addressed all of my concerns and done an excellent job on the revisions with new experimental data. I was also impressed with their response to other two reviewers. The paper is a significant contribution to the field.


** See Nature Research's author and referees' website at [www.nature.com/authors](http://www.nature.com/authors) for information about policies, services and author benefits


**COVID 19 and impact on peer review**
*As a result of the significant disruption that is being caused by the COVID-19 pandemic we are very aware that many researchers will have difficulty in meeting the timelines associated with our peer review process during normal times. Please do let us know if you need additional time. Our systems will continue to remind you of the original timelines but we intend to be highly flexible at this time.*

This email has been sent through the Springer Nature Tracking System NY-610A-NPG&MTS

Confidentiality Statement:

This e-mail is confidential and subject to copyright. Any unauthorised use or disclosure of its contents is prohibited. If you have received this email in error please notify our Manuscript Tracking System Helpdesk team at [*http://platformsupport.nature.com*](http://platformsupport.nature.com) .

Details of the confidentiality and pre-publicity policy may be found here [*http://www.nature.com/authors/policies/confidentiality.html*](http://www.nature.com/authors/policies/confidentiality.html)

[Privacy Policy](http://www.nature.com/info/privacy.html) | [Update Profile](https://mts-ncomms.nature.com)
DISCLAIMER: This e-mail is confidential and should not be used by anyone who is not the original intended recipient. If you have received this e-mail in error please inform the sender and delete it from your mailbox or any other storage mechanism. Springer Nature America, Inc. does not accept liability for any statements made which are clearly the sender's own and not expressly made on behalf of Springer Nature America, Inc.  or one of their agents.
Please note that neither Springer Nature America, Inc. or any of its agents accept any responsibility for viruses that may be contained in this e-mail or its attachments and it is your responsibility to scan the e-mail and attachments (if any).

**Reviewer comments, third version:**

**Author rebuttal, third version:**
